# Physical extraction of antigen and information

**DOI:** 10.1073/pnas.2320537121

**Published:** 2024-09-20

**Authors:** Hongda Jiang, Shenshen Wang

**Affiliations:** ^a^Department of Physics and Astronomy, University of California, Los Angeles, CA 90095

**Keywords:** adaptive immunity, active mechanics, Fisher information, selection fidelity, affinity discrimination

## Abstract

To respond and adapt, cells use surface receptors to sense environmental cues. While biochemical signal processing inside the cell is studied in depth, less is known about how physical processes during cell–cell contact impact signal acquisition. New experiments found that fast-evolving immune B cells in germinal centers (GCs) apply force to acquire antigen clusters prior to internalization, suggesting adaptive benefits of physical information extraction. We present a theory of stochastic antigen transfer and show that maximizing information gain via physical extraction can explain the dramatic phenotypic transition from naive to GC B cells—attenuated receptor signaling, enhanced force usage, and decentralized contact architecture. Our model suggests that binding-lifetime measurement and physical extraction serve as complementary modes of antigen recognition, greatly extending the dynamic range of affinity discrimination when combined. This physical-information framework further predicts that the optimal size of receptor clusters decreases as affinity improves, rationalizing the use of a multifocal synaptic pattern seen in GC B cells. By linking extraction dynamics to selection fidelity via discriminatory performance, we propose that cells may physically enhance information acquisition to sustain adaptive evolution.

## Significance

To sense and adapt, cells must capture and internalize external signals. Whereas information flow inside the cell has caught much attention, signal acquisition via cell–cell interfaces is less examined. Motivated by antigen extraction by immune cells using active mechanics, we present a physical-information framework of stochastic antigen transfer, which explains why receptor signaling gives way to physical extraction as cells are subject to natural selection for higher antigen-binding affinities. Our framework reveals complementary recognition modes, establishes a quantitative link from information gain to selection fidelity, and yields falsifiable predictions including optimal contact patterns. Altogether, our work elucidates the physical basis for persistent evolution of humoral immunity from an information perspective.

To move, respond, adapt, and communicate, cells use surface receptors to detect environmental signals, be they of chemical (1–3), mechanical (4–6), or thermal (7, 8) nature. Subsequent information processing inside the cell relies on networks of molecular interactions and chemical reactions. For this reason, a large body of theoretical work has aimed to understand the structure–function relationship of intracellular networks, by discovering functional motifs (9, 10), decoding the logic of decision-making (11, 12), exploring the design of feedback loops (13), and identifying the role of biological noise (14).

Physical processes occurring at the cell surface during sensing have received less attention. Originated from the seminal work of Berg and Purcell (15), an interesting line of research (16–20) quantified physical limits of sensing accuracy, set by the diffusive arrival of ligand molecules at receptors and stochasticity of their binding/unbinding kinetics. Parallel efforts were made to estimate energy cost of information processing and to determine when free energy dissipation can overcome equilibrium limits to sensory performance (21–24). However, past models have a number of restrictions: First, the sensory task is primarily to estimate extracellular chemical concentrations. Moreover, nonequilibrium processes downstream of receptor–ligand binding do not exert backward action. Often, sensory performance is not connected to functional outcomes.

New experiments revealed that B lymphocytes crucial for adaptive immunity employ a distinct, active route of signal acquisition prior to intracellular processing (25). Clonal expansion is an essential feature of immune responses. B cells bearing antigen-specific surface receptors (BCRs, 10^4^–10^5^ copies per cell) undergo this process in germinal centers (GCs)—microenvironments within organized lymphoid tissues in which B cells responses to antigen are amplified and refined in specificity (26). Through iterative cycles of cell division, somatic hypermutation, and selection, GC B cells expressing mutated BCRs with high affinity are positively selected. This rapid Darwinian-like evolution known as affinity maturation (27) produces protective antibodies and generates immune memory. B cell selection is a discriminatory task: Helper T cells discern among B cell clones and provide competitive survival signals according to their surface density of presented antigen (28). Ideally, B cells with higher affinity for antigen shall obtain, process, and present greater amounts of antigen in a given period of time and outcompete lower-affinity B cells for preferential T cell help. Strikingly, GC B cells acquire antigen from the surface of antigen-presenting cells (APCs) via an intensely physical mode: They gather receptor-bound antigens into dispersed clusters and extract them using internally generated contractile forces (29). Furthermore, as the B cell–APC interface becomes mechanically active, BCR signaling is attenuated (30, 31). It remains unclear why biochemical signaling gives way to physical sensing as cells begin to evolve. Can active force exertion make B cell affinities easier to discern? If so, does it ensure more faithful selection?

Adaptive evolution requires efficient selection which in turn necessitates an ability to distinguish receptor quality over a wide dynamic range. We hypothesize that physically modulating signal extraction can enhance information gain (i.e. distinguishability) for efficient adaptation. To test it, we formulate a biophysical model of stochastic antigen extraction and evaluate the information content of plausible readouts of BCR affinity. We find that binding-lifetime measurement and physical extraction represent complementary modes of antigen recognition, each achieving an optimal performance in a different affinity regime. When combined, they substantially expand the discrimination range of receptor quality.

Further, our theory identifies an upper bound for selection fidelity in terms of acquired information, revealing a direct impact of signal extraction on adaptive evolution. Notably, the antigen–tether strength constrains the range of distinguishable BCR affinities (32, 33). Our model predicts, therefore, for a fixed number of receptors, segregating into smaller clusters is optimal for discriminating higher affinities, contrary to the expectation that larger clusters would be more selective.

Therefore, by quantitatively connecting nonequilibrium extraction of antigen and information, our study identifies an active physical mode of biological sensing and recognition. Our results explain the striking phenotype switch—simultaneous changes in contact pattern, force usage and receptor signaling—that occurs when cells embark on rapid evolution. By linking information gain to selection fidelity, we propose that cells may physically enhance their distinguishability to support adaptive evolution.

## Physical-Information Framework of Antigen Extraction

**Fig. 1.**
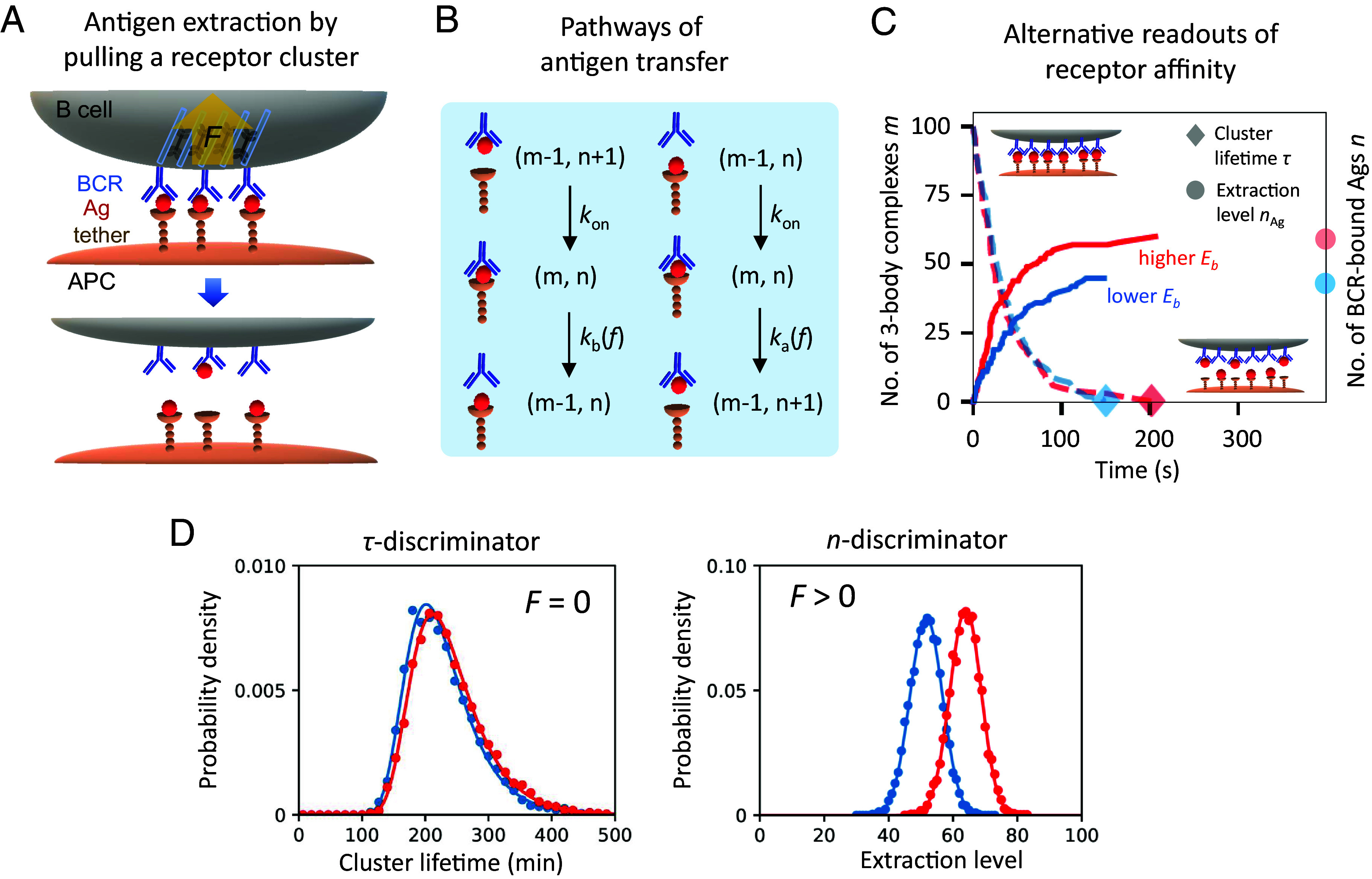
Physical extraction of antigen can improve discrimination of receptor affinity based on noisy readouts. (*A*) Schematic of a B cell applying force to extract antigen (Ag) tethered to the antigen-presenting cell (APC). Pulling force *F* is evenly distributed across bound complexes in a cluster. Each BCR–Ag–tether complex may break at either binding interface, leading to successful or futile antigen extraction. When all 3-body complexes break, a cluster dissociates and an extraction attempt ends. (*B*) Two pathways of antigen transfer: from APC to B cell (*Right*) and back (*Left*). Stochastic reactions occur one at a time to alter the system state characterized by the number of 3-body complexes, *m*, and the number of BCR-bound antigens, *n*. The *Upper* two reactions lead to a transition into the 3-body bound state at a rate $k_{\mathrm{on}}$ and the *Lower* two reactions result in a transition out of the 3-body bound state at a rate *k*_*a*_ or *k*_*b*_. The off-rate *k*_*a*_ (*k*_*b*_) depends on the intrinsic free energy barrier *E*_*a*_ (*E*_*b*_) and bond extension *x*_*a*_ (*x*_*b*_) corresponding to bond rupture as well as pulling force per complex of magnitude *f* (Eq. **2**). (*C* and *D*) Ensembles of extraction trajectories under similar BCR affinities (panel *C*, one trajectory from each ensemble) result in overlapping distributions of affinity readouts (panel *D*, *Left*: cluster lifetime, *Right*: extraction level). (*C*) Simulated trajectories depict time evolution of *m* (dashed) and *n* (solid) under modest pulling force (10 pN per complex). Red (blue) indicates higher (*Lower*) affinity. Symbols mark affinity readouts at cluster dissociation. (*D*) Physical extraction (*n*-discriminator) under pulling force outperforms lifetime measurement (*τ*-discriminator) without force in distinguishing high affinities ($E_{b}=10\;k_{B}T$ vs. $10.5\;k_{B}T$) based on noisy readouts. Each symbol is an average over 5,000 simulations. Curves are analytical results (Eq. **6**). Other parameters: *N* = 100, $E_{a}=8k_{B}T$, $x_{a}=1.5$ nm, and $x_{b}=2$ nm, $k_{\mathrm{on}}=0$. Independent complexes.

During antigen recognition, physical dynamics at the cell–cell interface are driven out of equilibrium: Active stress generated inside the B cell couples spatiotemporal organization of contact patterns to signal extraction. We choose to focus on the extraction stage, assuming that a pattern with BCR-bound antigen clusters preforms. By introducing a physical-information framework of antigen (Ag) extraction, we compute information content of alternative readouts of BCR–Ag binding affinity, resulting from the physical process of antigen transfer, that is, dissociation of antigens from the surface of APCs under active pulling forces (Fig. 1*A*). We consider vertical force as it may stabilize a multifocal contact pattern (34).

A distinctive feature of our model is the inclusion of antigen tethers; it creates a biologically realistic scenario in which BCR–Ag binding lifetime is measured in comparison to the finite duration of Ag–tether association, both subject to pulling stress. Force affects the kinetics of competitive bond dissociation and alters the likelihood of antigen extraction (32, 33). We demonstrate below that considering a finite tethering strength is crucial for understanding why cells switch from lifetime measurement to physical extraction and when distributed (multifocal) signal acquisition is favorable.

### Physical Extraction of Antigen Clusters.

We consider individual clusters of BCR–Ag–tether 3-body complexes as sensing units, each of size *N* to begin with. Within a cluster, parallel reactions of bond rupture and rebinding occur under a tugging force, as actomyosin assemblies pull on the cluster from inside the B cell (Fig. 1*A*, *Top*). The fact that fluctuations can cause instability (cluster dissociation) even if a mean-field analysis predicts stable attachment (a steady cluster) highlights the need for a stochastic description. Starting from maximum bond formation (with all *N* complexes being in the bound state), stochastic dynamics of antigen extraction follow a set of one-step master equations:

[1] $$\begin{array}{cc} dP_{m,n}\left(t\right)/dt= & [\left(\xi^{1,-1}-1\right)mk_{a}+\left(\xi^{1,0}-1\right)mk_{b}\\ & +\left(\xi^{-1,1}-1\right)nk_{\text{on}}\\ & +\left(\xi^{-1,0}-1\right)\left(N-m-n\right)k_{\text{on}}]P_{m,n}\left(t\right) \end{array}$$

Here, $P_{m,n}$ denotes the probability of having *m* 3-body complexes and a total of *n* BCR-bound antigens; it evolves over time due to dissociation of a closed bond at a rate of *k*_*a*_ (*k*_*b*_) if it occurs to the Ag–tether (BCR–Ag) interaction and formation of a new bond at a rate $k_{\mathrm{on}}$ on either side of the Ag (four terms on the right-hand side of Eq. **1**, illustrated in Fig. 1*B*). The step operator $\xi^{i,j}$ acts on a function $f_{m,n}$ according to $\xi^{i,j}f_{m,n}=f_{m+i,n+j}$, where i,j∈{±1}; it specifies the connectivity of the state space. Once all complexes in a cluster break, an attempt of antigen extraction ends and the cell locally detaches. We thus place an absorbing boundary at *m* = 0 (Fig. 1*C*, symbols marking cluster lifetime *τ* and extraction level $n_{\mathrm{Ag}}$ that are read out at cluster dissociation).

A key model ingredient is the tug-of-war setting of signal acquisition (25, 32, 33, 35) in which kinetics of competitive rupture between the Ag–tether (tethering) and BCR–Ag (tugging) interactions govern the likelihood and speed of antigen extraction (*SI Appendix*). We use Bell’s phenomenological model (36) to relate the off-rates to intrinsic bond properties (binding energy *E*_*i*_ and bond length *x*_*i*_; i=a,b) and pulling strength *f*:

[2] $$\begin{array}{c} \begin{array}{cc} & k_{a}\left(E_{a},x_{a},f\right)=k_{0}e^{-\beta (E_{a}-fx_{a})}=k_{a0}e^{\beta fx_{a}}\\ & k_{b}\left(E_{b},x_{b},f\right)=k_{0}e^{-\beta (E_{b}-fx_{b})}=k_{a}e^{-\beta \Delta E} \end{array} \end{array}$$

Here, *E*_*a*_ and *E*_*b*_ are the free energy barriers associated with rupture of the Ag–tether and BCR–Ag bonds, respectively, whereas *x*_*a*_ and *x*_*b*_ are the corresponding bond rupture lengths (36) that inversely relate to bond stiffness. *f* denotes the magnitude of force per 3-body complex. Our model encompasses two biologically plausible scenarios: independent complexes (f=const., i.e., total force scales with the instantaneous cluster size) and cooperative receptors (f=F/m, i.e., a fixed load *F* is evenly shared among *m* remaining complexes). $k_{a0}^{-1}=k_{0}^{-1}e^{\beta E_{a}}$ sets the time unit and energies are scaled by $1/\beta =k_{\text{B}}T$. The ratio between off-rates, $k_{b}/k_{a}$, is solely dependent on the effective BCR affinity $\Delta E=E_{b}-E_{a}-f\left(x_{b}-x_{a}\right)$. Note that only bonds in 3-body complexes are subject to pulling force such that their dissociation is exponentially faster than bond dissociation in 2-body complexes. We thus neglect antigen loss due to the latter over the timescale of extraction.

In principle, Kramers theory (37) provides a more accurate relationship between bond lifetime and applied force, as force not only lowers the free energy barrier of bond rupture but also deforms the landscape, displacing both the bound state and the transition states. However, Bell’s model offers a minimal description of the observed slip-bond behavior (29, 38), capturing the leading-order effect of physiological force on shortening bond lifetimes. Moreover, as we showed earlier (32, 33), Bell’s model recapitulates a functional characteristic of evolving B cells—stronger pulling reduces antigen extraction against stiff tethers (35). A refined description of reaction rates will not alter the qualitative results (*SI Appendix*).

### Affinity Readouts and Recognition Modes.

Cluster dissociation produces two natural readouts of receptor quality that can be measured for affinity discrimination among B cells: cluster lifetime and extraction level (the amount of extracted antigen); see Fig. 1*C*. Their respective distributions represent alternative outputs of stochastic extraction dynamics (Fig. 1*D*). Binding lifetime is a major determinant of self–nonself distinction made by cytotoxic T cells (39, 40). Meanwhile, receptor clustering serves as a means of kinetic proofreading (41–44). Activation of naive and memory B cells likely employs this canonical mode of affinity discrimination. On the other hand, antigen extraction level of evolving B cells is found to strongly correlate with their clonal reproductive fitness (28); B cell clones capable of acquiring and presenting larger amounts of antigen compete better for limited T cell help and are more likely to expand.

We refer to cells that perform these two modes of antigen recognition as *τ*-discriminator (lifetime measurement) and *n*-discriminator (physical extraction), respectively. We propose that physical extraction under pulling force outperforms lifetime measurement without force in distinguishing high affinities based on noisy readouts (Fig. 1*D*). To investigate this hypothesis, we address to what extent these measurements are informative of receptor quality, determine under what conditions information extraction is effective over a wide dynamic range, and explore ways in which physical dynamics of antigen extraction modulate information acquisition.

### Discrimination Performance.

Positive selection of higher affinity clones relies on faithful discrimination and ranking of receptor quality based on affinity readouts. Due to stochasticity in receptor–antigen kinetics, a cell with higher affinity receptors may produce a lower readout from antigen extraction, causing discrimination errors and unreliable ranking during selection. Hence, for a readout *Y*, representing *τ* or *n* hereafter, we define selection fidelity *ξ*_*Y*_ as the probability that a clone with higher affinity than its competitor produces a larger readout and thereby has a selective advantage. Provided two clones with affinities *E*_*b*_ and $E_{b}+\epsilon$, respectively, selection fidelity is given by

[3] $$\begin{array}{c} \begin{array}{c} \xi_{Y}=\int_{0}^{\infty }dy_{2}P_{Y}\left(y_{2};E_{b}+\epsilon \right)\int_{0}^{y_{2}}dy_{1}P_{Y}\left(y_{1};E_{b}\right), \end{array} \end{array}$$

where $P_{Y}\left(y;E_{b}\right)$ represents the readout distribution given BCR affinity *E*_*b*_. Intuitively, selection fidelity is limited by distinguishability between readout distributions with similar affinities. Fisher information (FI) is thus well suited for characterizing the discrimination performance of a recognition mode (*τ*- vs. *n*-discriminator), as it measures the “distance” between distributions with a small difference in an underlying parameter. We thus expect that, in the context of B cell selection, Fisher information builds a bridge from measurement (affinity readout) to function (selection fidelity).

Specifically, FI contained in the distribution $P_{Y}\left(y;E_{b}\right)$ of readout *Y* given BCR affinity *E*_*b*_ is defined by

[4] $$\begin{array}{c} \begin{array}{c} I_{Y}=\int {\left(\frac{d\;\ln\;P_{Y}\left(y;\;E_{b}\right)}{dE_{b}}\right)}^{2}P_{Y}\left(y;E_{b}\right)dy \end{array} \end{array}$$

which evaluates the mean square gradient of the log-likelihood with respect to *E*_*b*_. FI is large if a small variation of *E*_*b*_ leads to a considerable change in the readout distribution, so that a cell can make an accurate estimate of its receptor quality from measuring the readout *Y*.

To make the intuition that selection fidelity is limited by discrimination accuracy a quantitative statement, we seek a universal upper bound of *ξ*_*Y*_. In the hard-discrimination regime (with $\epsilon \ll E_{b}$), we find that

[5] $$\begin{array}{c} \begin{array}{c} \xi_{Y}\leq \mu_{\mathrm{CDF}}+\sigma_{\mathrm{CDF}}\sqrt{I_{Y}}\epsilon +o\left(\epsilon^{2}\right) \end{array} \end{array}$$

Here, $\mu_{\mathrm{CDF}}$ and $\sigma_{\mathrm{CDF}}^{2}$ are respectively the mean and variance of the cumulative distribution function of readout *Y*. Notably, the square-root scaling of the fidelity bound with the information content measured by $I_{Y}$ remains the same, regardless of the number of distinct clones or the specific readout distribution (*SI Appendix*). In fact, a similar distinguishability bound in the form of $\sqrt{I_{Y}}$ was found in neuronal population coding (45).

Indeed, connections between information and precision of measurements based on thermodynamic processes are widely drawn in the context of cellular sensing and computation (21–24, 46–48). What is different here is that our framework casts the nonequilibrium process of antigen extraction by rapidly evolving cells into a form of physical extraction of information used for adaptation. As we show below, through physically altering the extraction dynamics, cells can actively modulate the nature and statistics of readout distributions, making them easier to distinguish. This enhancement of information acquisition in turn permits more faithful selection and facilitates persistent adaptation.

## Results

### *τ*-Discriminator and *n*-Discriminator Perform Optimally in Different Affinity Regimes.

To attain analytical intuition on physical information extraction, we begin with the simplest case of independent 3-body complexes (f= const.) with negligible rebinding ($k_{\mathrm{on}}=0$). With *N* independent extraction events, the extraction level $n_{\mathrm{Ag}}$ simply follows a binomial distribution, whereas the cluster lifetime *τ* is set by the longest-lived complex and thereby following an extreme value distribution. We thus have

[6] $$\begin{array}{c} \begin{array}{c} P\left(\tau \right)=N\lambda e^{-\lambda \tau }{\left(1-e^{-\lambda \tau }\right)}^{N-1},n_{\mathrm{Ag}}∼B\left(N,\eta \right). \end{array} \end{array}$$

The lifetime distribution depends on the off-rate of a 3-body complex, $\lambda =k_{a}+k_{b}$, while the single-complex extraction probability $\eta =k_{a}/\left(k_{a}+k_{b}\right)$ determines the distribution of extraction level. These distributions yield the mean cluster lifetime $\mu_{\tau }\equiv \left\langle \tau \right\rangle \approx \;\ln N/\lambda$ and the mean extraction level $\mu_{n}\equiv \left\langle n_{\mathrm{Ag}}\right\rangle =N\eta$, with variances $\sigma_{\tau }^{2}\equiv \left\langle {\left(\tau -\mu_{\tau }\right)}^{2}\right\rangle \approx \pi^{2}/\left(6\lambda^{2}\right)$ and $\sigma_{n}^{2}\equiv \left\langle {\left(n_{\mathrm{Ag}}-\mu_{n}\right)}^{2}\right\rangle =N\eta \left(1-\eta \right)$, respectively.

**Fig. 2.**
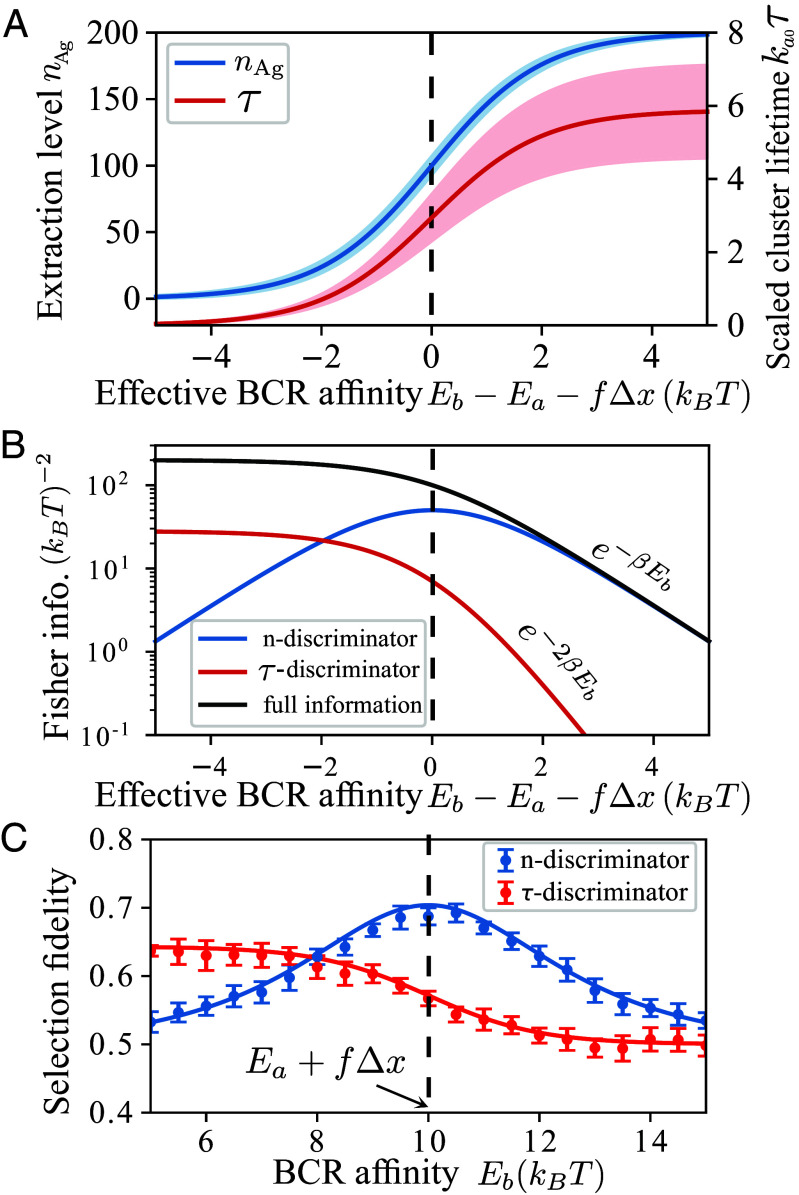
*n*-discriminator and *τ*-discriminator perform optimally in different affinity regimes. (*A*) Affinity readouts as a function of effective BCR affinity. Solid lines indicate the mean and the shades *Above* and *Below* delineate one SD, calculated from Eq. **6**. Cluster lifetime is scaled by $1/k_{a0}=e^{\beta E_{a}}/k_{0}$. (*B*) Fisher information encoded in cluster lifetime (red, $I_{\tau }$ from Eq. **7**), antigen extraction level (blue, $I_{n}$ from Eq. **7**), and the entire dissociation trajectory (black, $I_{\mathrm{full}}$ from Eq. **8**). As BCR affinity increases, information content of cluster lifetime decays much faster than that of extraction level. (*C*) Selection fidelity between B cells with a small affinity difference $\epsilon =0.1\;k_{B}T$. Each symbol is an ensemble average over 2,000 Gillespie simulations of Eq. **1** and the error bar is due to 10 independent realizations of the ensemble. Curves depict the *Upper* bound in relation to Fisher information (Eq. **5**). Vertical dashed lines mark the condition of vanishing affinity gap under force, i.e., $E_{b}=E_{a}+f\Delta x$. Independent complexes, each subject to a constant force of magnitude *f*, no rebinding. $N=200,x_{a}=1.5\mathrm{nm},x_{b}=2\mathrm{nm},E_{a}=9\;k_{B}T$, and $f\Delta x=k_{B}T$.

Fig. 2*A* shows that, as receptor affinity *E*_*b*_ increases, both readouts increase on average, in line with the observation that B cells of higher affinity form a longer contact with the APC and acquire a larger amount of antigen (49). Nevertheless, both readouts reach saturation at high affinities, indicating the existence of a limited tether strength. Saturation occurs once the affinity of receptor–antigen-binding well exceeds that of antigen–tether attachment. At this point, rupture predominantly occurs at the tether bond, rendering further enhancement in receptor affinity incapable of increasing cluster lifetime or extraction level ($\lambda \approx k_{a},\eta \approx 1$). In fact, our recent analysis (33) suggests that a limited tether strength under tugging force might be the physical origin of a modest affinity ceiling for in vivo B cell evolution (50), far below the maximum affinity of antibody mutants evolved in vitro (51).

We now compare how well the two modes of antigen recognition can distinguish receptor quality based on noisy readouts. Using Eq. **4** to calculate Fisher information $I_{Y}$ from readout distributions $P_{Y}\left(y;E_{b}\right)$ given by Eq. **6**, we obtain (*SI Appendix*)

[7] $$\begin{array}{c} \begin{array}{c} I_{\tau }\approx \beta^{2}\frac{{\left(\ln N\right)}^{2}}{{\left(1+e^{\beta \Delta E}\right)}^{2}},I_{n}=\beta^{2}\frac{Ne^{\beta \Delta E}}{{\left(1+e^{\beta \Delta E}\right)}^{2}}, \end{array} \end{array}$$

where $\Delta E=E_{b}-E_{a}-f\left(x_{b}-x_{a}\right)$ is the effective BCR affinity—the difference between the receptor–antigen (*E*_*b*_) and antigen–tether (*E*_*a*_) binding energies under pulling force *f*. For a stiff APC ($x_{a}<x_{b}$), the effective BCR affinity is low when force per bond *f* is large. Interestingly, $I_{\tau }$ and $I_{n}$ exhibit distinct dependence on effective affinity ΔE (Fig. 2*B*) and scale differently with the initial cluster size *N* (*SI Appendix*, Fig. S1).

First, $I_{\tau }$ decreases monotonically with increasing effective affinity (Fig. 2*B*, red curve). This is because measurements of complex lifetime are informative of receptor affinity only if a large number of rupture events occur on the receptor side ($\lambda \approx k_{b}$); this becomes less likely as receptor affinity increases. In contrast, $I_{n}$ peaks at intermediate affinities (Fig. 2*B*, blue curve). This reflects that the Bernoulli process of antigen extraction is most sensitive to affinity changes when receptor–antigen lifetime matches antigen–tether lifetime, i.e., when the affinity gap closes (ΔE=0, η=1/2).

In addition, $I_{n}$ exceeds $I_{\tau }$ at high affinities and decays more slowly with increasing *E*_*b*_: $e^{-\beta E_{b}}$ vs. $e^{-2\beta E_{b}}$. This difference stems from the distinct affinity dependence of fluctuations in readout measurements, as shown by the color shades in Fig. 2*A*. The accuracy of lifetime measurements is limited by noise in tether lifetime and, therefore, the uncertainty *σ*_*τ*_ remains finite even if receptor affinity reaches a high value ($\sigma_{\tau }∼1/\lambda \to 1/k_{a}$; red shade in Fig. 2*A*). By contrast, the variance of extraction level, $\sigma_{n}^{2}=N\eta \left(1-\eta \right)$, vanishes as the extraction probability $\eta ={\left(1+k_{b}/k_{a}\right)}^{-1}$ approaches 1 at high affinities ($k_{b}/k_{a}\to 0$, blue shade in Fig. 2*A*). Simply put, cluster lifetime measures the (inverse) sum of off rates and is only informative of low receptor affinities, whereas extraction level measures the ratio of off rates and hence remains informative even at high receptor affinities. Therefore, two recognition modes operate optimally in different affinity regimes and would allow accurate discrimination over a wide dynamic range when combined.

Furthermore, $I_{\tau }$ shows a weaker scaling with the initial cluster size *N* than $I_{n}$ does, i.e., $I_{\tau }∼{\left(\ln N\right)}^{2}$ and $I_{n}∼N$ (*SI Appendix*, Fig. S1, panel *A* vs. *C*, black curve), implying that $I_{n}$ rapidly exceeds $I_{\tau }$ as *N* grows. This behavior can be understood from how individual rupture events contribute to the final readout at cluster dissociation. As a cluster dissociates via successive rupture of independent complexes, most of the time is spent on the last few events (*SI Appendix*, Fig. S2*A*, gray histogram), resulting in low statistics and high noise in cluster lifetime. In contrast, $I_{n}$ is linear in *N*, because all complexes contribute to extraction equally and independently.

We next evaluate how the information content of these readouts compares with the full information encoded in the entire extraction trajectory. Each trajectory comprises two concurrent sequences: a sequence of waiting times between successive reactions, $\left\{t_{1},\cdots ,t_{i},\cdots ,t_{N}\right\}$ and a sequence of reaction types, $\left\{s_{1},\cdots ,s_{i},\cdots ,s_{N}\right\}$, where *s*_*i*_ indicates whether the rupture event occurs at the receptor side or the tether side. Without rebinding, the extraction trajectory corresponds to a cascade of *N* rupture events. One can compute the full information $I_{\mathrm{full}}$ from the probability of observing each possible trajectory $P(\left\{t_{1},s_{1};t_{2},s_{2};...;t_{N},s_{N}\right\})$ (*SI Appendix*) and obtain

[8] $$\begin{array}{c} \begin{array}{c} I_{\mathrm{full}}=I_{\left\{t_{i}\right\}}+I_{\left\{s_{i}\right\}}=\beta^{2}\frac{N}{1+e^{\beta \Delta E}}. \end{array} \end{array}$$

One immediately sees that $I_{n}\to I_{\mathrm{full}}$ at large values of ΔE (Fig. 2*B*, blue and black curves). In other words, at high affinities, extraction level contains nearly complete information about receptor affinity obtainable from extraction trajectories, because, for independent complexes, the order of rupture events contains no information about receptor affinity. While extraction level preserves information encoded in the sequence of reaction types ($I_{n}=I_{\left\{s_{i}\right\}}$), cluster lifetime *τ* ($=\sum_{i=1}^{N}t_{i}$) loses information contained in the distribution of waiting times. Thus, $I_{\tau }<I_{\left\{t_{i}\right\}}$ already for modest receptor affinities (Fig. 2*B*, red vs. black curves).

Finally, to determine whether the information value of affinity readouts imposes an upper bound to selection fidelity, we compare the theoretical bound (Eq. **5**) to fidelity obtained from simulations of antigen extraction by cells with similar affinities. Fig. 2*C* shows that the theoretical bound (curve) agrees well with the simulated fidelity (symbols) over a wide affinity range. Furthermore, the simulated fidelity follows the same affinity dependence as Fisher information (color curves in Fig. 2*B* vs. *C*), confirming that selection fidelity is indeed bounded by distinguishability of nearby readout distributions.

### Role of Cooperativity via Load Sharing.

Antigen transfer between cells occurs through synaptic contact, where antigen-bound receptors phase separate from adhesion molecules to form distributed units for information processing (29, 52). We first study information extraction via one such reaction unit and then identify the optimal cluster size that maximizes total information acquisition through multifocal extraction (Section C). One natural scenario in which 3-body complexes couple within a cluster is through sharing contractile stress exerted by the actomyosin machinery that assembles underneath the B cell membrane and pulls on antigen-bound BCR clusters (29, 52). Assuming that a constant force *F* acts uniformly across a cluster, extraction dynamics depend on the instantaneous cluster size *m* via the off rates:

[9] $$\begin{array}{c} \begin{array}{c} k_{a,m}=k_{0}e^{-\beta (E_{a}-\frac{F}{m}x_{a})},\;\;k_{b,m}=k_{0}e^{-\beta (E_{b}-\frac{F}{m}x_{b})}. \end{array} \end{array}$$

As more bonds break, the force F/m on each remaining complex increases, resulting in accelerated dissociation.

Coupling and nonlinearity introduced by load sharing make the calculation of the exact FI from the full readout distribution (Eq. **4**) no longer analytically tractable. Fortunately, a general lower bound, ${\tilde{I}}_{Y}$, provides an extremely good approximation of the exact FI and reduces the calculation to finding the first two moments of the readout distribution (53):

[10] $$\begin{array}{c} \begin{array}{c} I_{Y}\geq {\tilde{I}}_{Y}\equiv \frac{1}{\sigma_{Y}^{2}}(\frac{d\mu_{Y}}{dE_{b}}{)}^{2}. \end{array} \end{array}$$

Here, *μ*_*Y*_ and *σ*_*Y*_ are respectively the mean and SD for readout *Y*. In fact, this lower bound shows an excellent agreement with the exact FI even for modest clusters (*SI Appendix*, Fig. S1, symbols falling on curves); see *SI Appendix* for details of iterative calculations of the moments for both readouts.

**Fig. 3.**
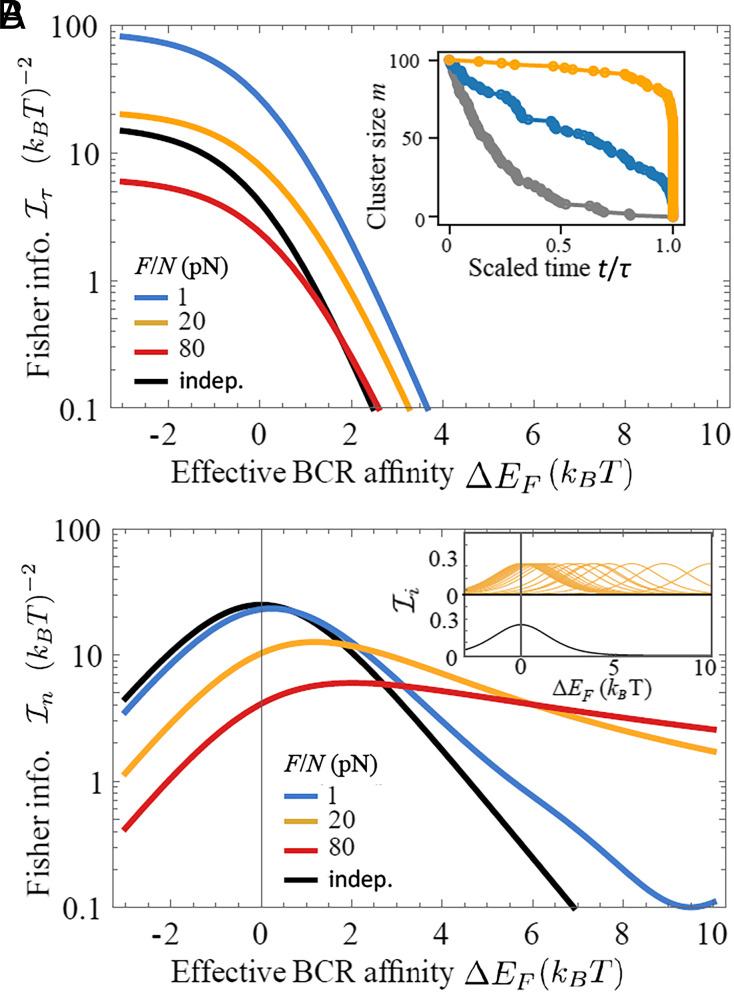
Cooperativity due to load sharing can enhance information extraction. Fisher information as a function of effective BCR affinity $\Delta E_{F}=E_{b}-E_{a}-F\Delta x/N$ is shown under varying force magnitude *F*. The black curve corresponds to independent complexes (i.e. force per complex remaining constant during cluster dissociation). (*A*) Fisher information retained in cluster lifetime, based on Eq. **11**. *Inset*: examples of dissociation trajectories for independent complexes (black) and under a shared load (blue: F/N=1 pN, orange: F/N=20 pN). (*B*) Fisher information contained in extraction level, obtained from Eq. **12**. *Inset*: information content of successive rupture events for *F* = 0 (black) and F/N=20 pN (orange). Sensitive windows centered at higher affinities represent contributions from rupture events occurring at later times, when fewer complexes remain bound and share the load. Parameters: $x_{a}=1.5\mathrm{nm},x_{b}=2\mathrm{nm}$, and *N* = 100.

Interestingly, this simple scenario of load sharing already exhibits nontrivial behavior. Unlike the case of constant force per bond where the cluster lifetime is set by the few long-lived complexes, sharing a moderate load accelerates rupture of the last few bonds, such that the majority of rupture events contribute nearly equally to the cluster lifetime, which consequently reduces the relative variance of *τ* and increases its information content (Fig. 3*A*). On the other hand, since individual complexes now break under different force magnitudes, the *n*-discriminator can distinguish a wider range of affinities compared to the case of independent complexes (Fig. 3*B*).

To illustrate these effects of load sharing, we first ignore rebinding. We evaluate Eq. **10** for a *τ*-discriminator, using the mean and variance of cluster lifetime, $\mu_{\tau }=\sum_{i=1}^{N}1/\left(i\lambda_{i}\right)$ and $\sigma_{\tau }^{2}=\sum_{i=1}^{N}1/{\left(i\lambda_{i}\right)}^{2}$, with $\lambda_{i}=k_{a,i}+k_{b,i}$ (*SI Appendix*). This gives

[11] $$\begin{array}{c} \begin{array}{c} {\tilde{I}}_{\tau }=\beta^{2}\frac{1}{\sum_{i=1}^{N}{\left(i\lambda_{i}\right)}^{-2}}{\left(\sum_{i=1}^{N}\frac{k_{b,i}}{i\lambda_{i}^{2}}\right)}^{2} \end{array} \end{array}$$

which depends on the effective BCR affinity $\Delta E_{i}=E_{b}-E_{a}-F\Delta x/i$ and on the pulling force *F* via $k_{a,i}$ and $k_{b,i}$. In Fig. 3*A* we show that sharing a small load (F/N=1pN, blue curve) can greatly increase information encoded in *τ* at relatively low BCR affinities, compared to independent extraction events under constant *f* (black curve). This can be understood from dissociation trajectories (Fig. 3*A*, *Inset*) and waiting-time distributions (*SI Appendix*, Fig. S2). Under very strong or very weak pulling per complex, cluster lifetime is predominantly set by the first or last few rupture events, respectively (Fig. 3*A*, *Inset*, orange and gray trajectories). Under intermediate forces, however, the vast majority of rupture events are almost equally spaced in time (*SI Appendix*, Fig. S2*A*, blue histogram), leading to a roughly linear decline in cluster size over time (Fig. 3*A*, *Inset*, blue trajectory). Thus, sharing a modest load alters the cluster lifetime distribution from extreme-value types to Gaussian-like (*SI Appendix*, Fig. S3*A*). As a result, the relative variance of cluster lifetime is minimal (*SI Appendix*, Fig. S4*B*) and the information content is maximal (*SI Appendix*, Fig. S3*A*) when complexes are coupled under modest pulling.

For an *n*-discriminator, cooperativity may act to expand the range of distinguishable affinities at the cost of discrimination stringency (Fig. 3*B*). Using the mean $\mu_{n}=\sum_{i=1}^{N}\eta_{i}$ and the variance $\sigma_{n}^{2}=\sum_{i=1}^{N}\eta_{i}\left(1-\eta_{i}\right)$, where $\eta_{i}=k_{a,i}/\left(k_{a,i}+k_{b,i}\right)$, we obtain the lower bound

[12] $$\begin{array}{c} \begin{array}{c} {\tilde{I}}_{n}=\sum_{i=1}^{N}I_{i}=\beta^{2}\sum_{i=1}^{N}\frac{e^{\beta \Delta E_{i}}}{{\left(1+e^{\beta \Delta E_{i}}\right)}^{2}} \end{array} \end{array}$$

Here, $I_{i}$ denotes information encoded in the reaction type when a total of *i* complexes remain closed. Total information ${\tilde{I}}_{n}$ sums over contributions from individual rupture events, $\left\{I_{i}\right\}$, each having its own sensitive window for affinity discrimination (*SI Appendix*, Fig. S5, red envelope and purple humps). $I_{i}$ peaks at $E_{b,i}^{*}=E_{a}+F\Delta x/i$, the most discernible affinity with *i* remaining complexes; $E_{b,i}^{*}$ is at the center of the corresponding sensitive window where the affinity gap $\Delta E_{i}$ closes (Fig. 3*B*, *UpperInset*). Note that the shared load *F* controls the spacing between adjacent windows. When force per complex is constant, all sensitive windows coincide (Fig. 3*B*, *LowerInset*) and ${\tilde{I}}_{n}$ is high within a narrow affinity range (Fig. 3*B*, black curve). In contrast, under a moderate shared load, adjacent sensitive windows become partially overlapping while together they cover a wide range of affinities (Fig. 3*B*, *UpperInset* and orange curve), allowing efficient information extraction as affinity improves through evolution. Too strong loading is counterproductive, because sensitive windows become well separated and total information remains low (Fig. 3*B*, red curve).

So far we have neglected the effect of rebinding on information acquisition. We show in *SI Appendix*, Text and Fig. S6 that frequent rebinding strongly enhances the performance of a *τ*-discriminator at a cost of extraction speed, but it has a very limited impact on an *n*-discriminator. In particular, rebinding does not alter the affinity dependence at high affinities; we still get $I_{\tau }\propto e^{-2\beta E_{b}}$ and $I_{n}\propto e^{-\beta E_{b}}$ when $\beta \Delta E_{i}\gg 1$. That is, physical extraction robustly outperforms lifetime measurement when it comes to distinguishing high affinities.

### Naive and Evolving Cells Favor Distinct Recognition Modes and Contact Patterns.

Recent experiments have revealed that B cells undergo a dramatic transformation of their phenotype when they enter the GC microenvironment that facilitates rapid somatic evolution: They alter their signaling pathways for cell activation, their cytoskeletal organization during antigen recognition, and their manner of force application all at once (29–31, 52). Curiously, only for evolving cells, BCR signal transduction is attenuated; meanwhile, the B cell–APC interface becomes mechanically active and coalesces receptor-bound antigens into clusters for physical extraction. It remains unknown why mechanical sensing overtakes receptor signaling when cells undergo selection for improved antigen recognition. We hypothesize that this transition allows selective expansion of higher affinity clones by enabling efficient and broad discrimination of receptor quality.

Consider a total of *N*_0_ complexes divided into clusters of size *N*, each subject to a pulling force of magnitude *F*; this accounts for the modular structure of actomyosin assemblies that constrains forces exerted on clusters. In this mean-field description with homogeneous *N* and *F* among clusters, the total information is simply

$$\begin{array}{c} I_{Y}^{\mathrm{tot}}=\frac{N_{0}}{N}I_{Y}\left(N,F\right) \end{array}$$

**Fig. 4.**
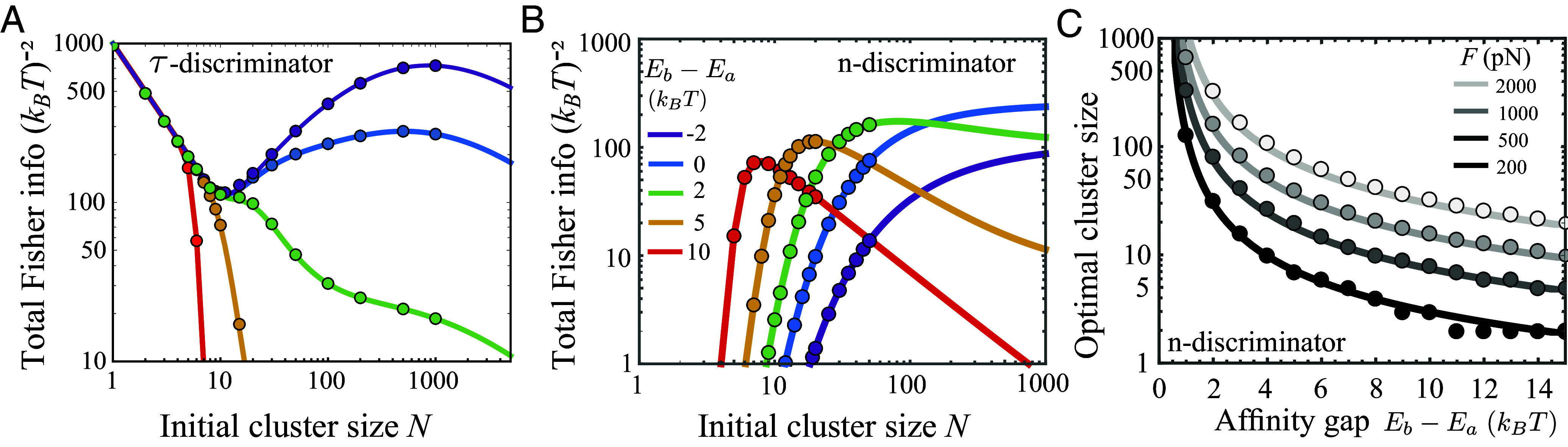
Optimal cluster size that maximizes total information depends on recognition mode and affinity regime. (*A* and *B*) Total Fisher information provided by *N*_0_ complexes that are equally divided into clusters of size *N*. Curves are *Lower* bounds of total Fisher information, $N_{0}{\tilde{I}}_{Y}/N$, calculated from the mean and variance of the readout (Eq. **10**). Symbols are exact values of Fisher information evaluated numerically using the readout distribution (Eq. **4**; see *SI Appendix* for details); this is only feasible up to moderately large clusters. We consider a constant shared load *F* = 500 pN acting on each cluster, without rebinding. Panels (*A* and *B*) display the total information acquired by a *τ*-discriminator and an *n*-discriminator, respectively. *τ*-discriminators use a large cluster to distinguish low affinities (purple), whereas *n*-discriminators form intermediate clusters to distinguish high affinities (red). (*C*) The optimal cluster size that maximizes the total information extracted by an *n*-discriminator decreases with increasing BCR affinity, as stronger force per complex is required to close the increasingly large affinity gap. Solid lines are approximations based on Eq. **13**. Symbols correspond to peak locations of the information curves, like those in panel (*B*). $x_{a}=1.5\mathrm{nm},\mathrm{and}x_{b}=2\mathrm{nm}$.

We find that, depending on the affinity regime, cells may employ distinct contact patterns and pulling strengths for maximal information extraction (Fig. 4). The key is that, changes in cluster size alter the initial force per complex, which in turn modulates the waiting time distribution and modifies the effective BCR affinity (Section B).

For a *τ*-discriminator, using independent complexes (*N* = 1) is favorable regardless of affinity (Fig. 4*A*, a common interception with the y-axis), due to a minimum loss of information from measurements of binding lifetime. Specifically, for small clusters under strong force per complex, cluster lifetime is dominated by the time until the first rupture event which almost always occurs on the receptor side. In this case, $I_{\tau }=\beta^{2}$ and hence $I_{\tau }^{\mathrm{tot}}=\beta^{2}N_{0}/N$, resulting in an affinity-independent 1/N decline of the total information (Fig. 4*A*, small-*N* regime). In words, fewer clusters of larger sizes yield fewer independent measurements of receptor affinity.

**Fig. 5.**
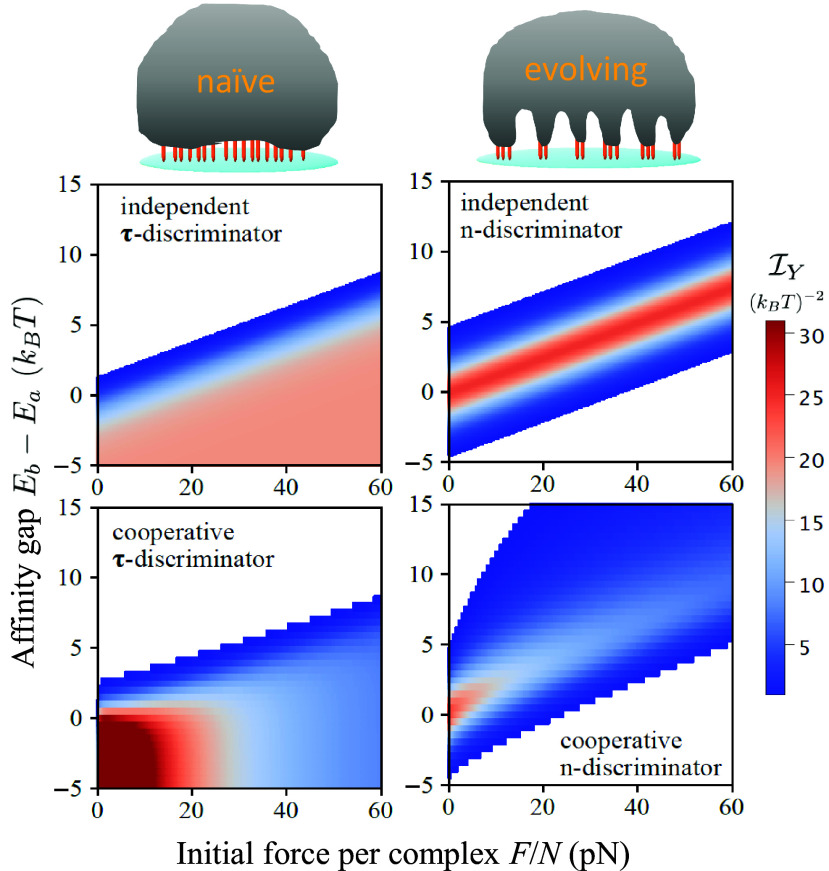
B cells may switch recognition mode to ensure broad discrimination and persistent adaptation. *Top*: schematic of distinct phenotypes (morphology and contact pattern) and recognition modes assumed by naive and evolving B cells, respectively. Orange bars represent coarse-grained receptor–antigen–tether complexes. *Left* column: *τ*-discriminator; *Right* column: *n*-discriminator. Color-coded FI is shown as a function of initial force per complex and affinity gap. *Upper* panels depict the exact FI (Eq. **4**) for independent extraction events (constant *f*). *Lower* panels display the *Lower* bound of FI (Eq. **10**) for cooperative extraction events under a shared load *F*. The white region indicates the regime where $I_{Y}<1\left[{\left(k_{B}T\right)}^{-2}\right]$, corresponding to indistinguishable affinities. *N* = 100, $x_{a}=1.5$ nm, $x_{b}=2$ nm, and $k_{\mathrm{on}}=0$.

Notably, at low affinities (e.g. purple curve), total information exhibits a nonmonotonic dependence on cluster size, with a near-optimal peak at a large cluster size. There, modest force per complex (F/N∼1pN) alters the nature of the waiting time distribution from exponential (large F/N) or an extreme-value type (vanishing F/N) to Gaussian-like, resulting in a large number of informative measurements. However, total information falls rapidly as receptor affinity increases (purple to red), because far fewer rupture events probe receptor–antigen binding. Thus, a *τ*-discriminator can distinguish weak affinities using large clusters under vanishing force per complex. This recognition mode is suited for naive cells prior to affinity maturation (Fig. 5, *Left* column).

In contrast, an intermediate cluster size maximizes the total information an *n*-discriminator obtains (Fig. 4*B*, peak in curves having $E_{b}>E_{a}$). As shown earlier, a force per complex that closes the affinity gap maximizes information gain for independent complexes. To determine the affinity dependence of the optimal cluster size under load sharing, we calculate the lower bound of total information, ${\tilde{I}}_{n}^{\mathrm{tot}}$, acquired through antigen extraction via a rupture cascade (*SI Appendix*). For a given affinity gap $\Delta E_{0}=E_{b}-E_{a}$, ${\tilde{I}}_{n}^{\mathrm{tot}}$ is a function of FΔx/N only. The optimal cluster size $N^{*}$ that maximizes ${\tilde{I}}_{n}^{\mathrm{tot}}$ satisfies

[13] $$\begin{array}{c} \begin{array}{c} \frac{F\Delta x}{N^{*}}\approx c\left(\Delta E_{0}\right)\Delta E_{0}, \end{array} \end{array}$$

where $c(\Delta E_{0})$ increases monotonically with $\Delta E_{0}$ and saturates at high affinities, i.e., $c(\Delta E_{0})\to 1$ for $\beta \Delta E_{0}\gg 1$. Thus, force per complex in an optimal cluster has a superlinear dependence on affinity gap, due to stronger forces exerted on complexes that break at later times. Eq. **13** agrees very well with numerical results (Fig. 4*C*). It suggests that, by adjusting the contact pattern such that F/N matches the affinity gap $\Delta E_{0}$, cells can maximize information extraction. A testable prediction thus follows: The optimal cluster size decreases as receptor affinity improves due to evolution (Fig. 4 *B* and *C*). Therefore, active antigen recognition via a multifocal pattern, as is indeed observed in evolving B cells (29, 52), may confer an adaptive benefit; this recognition mode, when coupled to a feedback mechanism that tunes the cluster size or force magnitude according to an increasing affinity, can potentially enhance affinity discrimination on the fly and promote expansion of newly produced potent clones (Fig. 5, *Right* column).

These results support our hypothesis that, by enabling broad affinity discrimination, a phenotypic transition permits persistent immune adaptation. This transition is characterized by switching from receptor signaling to active mechanics, forming decentralized contacts instead of a bull’s eye pattern, and gauging extraction efficiency rather than measuring binding lifetime. Importantly, our analysis suggests that cells maximize Fisher information via physical dynamics, which in turn yield functional behaviors. This finding demonstrates that distinguishability is not an intrinsic property of receptor–ligand binding, but depends on the measurement a cell performs on it. In this sense, not only that the device and bits of biological computation are physical, so is the execution process of information-handling algorithms.

## Discussion

We investigate how, and why, rapidly evolving immune cells estimate and rank their receptor affinity by physically acquiring antigens. This study is motivated by a dramatic phenotype transition from naive to GC B cells, and by the intuition that the fidelity of B cell selection is limited by the distinguishability of readout distributions with similar affinities. We present a physical-information framework to first compute affinity readouts resulting from antigen extraction, and then evaluate the discriminatory accuracy based on noisy data. Using this framework, we show that both the choice and the execution of affinity measurement matter. In particular, we find that information gain depends on force magnitude and contact pattern, which affect how antigen extraction occurs via pulling on receptor clusters. We use Fisher information to measure how sensitive the readout distributions are to changes in receptor affinity. Importantly, we identify an upper bound for selection fidelity in terms of Fisher information, which suggests that information gain indeed confers an adaptive benefit, since it increases the chance that a higher-affinity cell produces a larger readout and gets preferentially selected.

Indeed, mutual information is a classical metric for estimating information transmission. As a *global* measure of distinguishability, it has been applied to ligand classification of T lymphocytes (54). Fisher information, on the other hand, is suited for measuring affinity-dependent discrimination performance relevant to B cell selection; it represents a *local* measure of sensitivity, taking a differential form of the Kullback–Leibler divergence between nearby distributions in parameter space.

The simplicity of our model framework with only a few key ingredients—tug-of-war antigen extraction and state-dependent force application—permits predictive understanding of major phenomenology. First, lifetime measurement and physical extraction may represent complementary modes of antigen recognition and affinity discrimination (Fig. 2); the former is employed by naive B cells, whereas the latter is characteristic of evolving B cells. While cluster lifetime is informative of relatively low receptor affinity (relative to tether affinity), extraction level performs optimally when receptor and tether affinities match and degrades slowly as affinity increases further, thus greatly extending the discrimination range. Second, receptor coupling due to cytoskeletal loading influences two modes of information extraction in distinctive ways (Fig. 3). By altering the waiting time distribution from exponential to Gaussian-like, a modest load yields numerous informative measurements made by a *τ*-discriminator. On the other hand, preferred force magnitudes for an *n*-discriminator serve to close the affinity gap between receptor and tether bonds, yielding a maximal gain in distinguishability between similar BCR affinities. These results highlight the role of antigen tether in setting the reference affinity and limiting the range of discrimination. A falsifiable prediction thus follows: While *τ*-discriminators form a centralized pattern with a large receptor cluster to distinguish low affinities, *n*-discriminators adopt a multifocal pattern with intermediate cluster sizes that decrease with increasing BCR affinity (Fig. 4). This prediction can be tested by measuring the fluorescence intensity of receptor/antigen clusters formed in the synapse of B cells with a range of affinities.

Altogether, these findings rationalize the observed phenotype transition: As rapid evolution begins to operate, persistent adaptation demands efficient affinity discrimination over a wide dynamic range. Simultaneous changes in the synaptic pattern, force usage, and signaling pathway, as our model anticipates, suggest that maximizing information extraction for persistent adaptive evolution underlies the switch from lifetime measurement of naive cells to physical signal extraction of evolving cells (Fig. 5). Conceptually, our work focuses on information acquisition prior to intracellular processing. It therefore complements studies of biochemical circuitry downstream of signal internalization.

To carry out their effector or memory functions, B cells must differentiate into plasma cells or memory B cells, respectively. This fate decision is a complex phenomenon not fully understood. Although a recent study suggests that lower-affinity B cells are preferentially recruited to the memory pool (55), the mechanism by which affinity may promote either fate remains unclear and could depend on signals from both the BCR and T cell help. Moreover, factors other than affinity have been found to contribute to B cell fate choice, including differences in force sensitivity and signaling capability of BCR isotypes (IgM vs. IgG) (56), asymmetric cell division (an unequal distribution of fate-altering molecules between daughter cells) (57), as well as a temporal hypothesis—memory B cells are generated mostly in pre-GC and early GC periods, after which long-lived plasma-cell differentiation becomes more pronounced (58). Our theory suggests to examine potential differences in force usage, signaling pathway, and synaptic pattern between plasma cells and memory B cells, as a means to probe whether the principle of maximizing information extraction also applies to fate choice. Given that memory B cells appear to represent a more plastic phenotype (capable of GC reentry and further maturation), we anticipate that their behavior has a richer relationship to force and that force exertion may depend on affinity.

Synapse formation is a mechanism for locally amplifying antigen concentration even when antigen abundance is globally limited. As antigens degrade, get consumed, or decline due to infection clearance, antigen clusters formed by synaptic patterning will likely become smaller, thereby exerting a stronger selection pressure for improving BCR affinity. Our theory predicts that, as affinity increases, smaller clusters enhance information acquisition. That is, antigen limitation can be beneficial to maintaining discriminatory performance and facilitating efficient B cell selection. One way to test this prediction is by reducing the amount of antigen tethered to artificial substrates or live APCs and measuring extraction of GC B cells ex vivo. We expect enhanced discrimination, manifested as a stronger contrast in antigen extraction level between high and lower affinity B cells. Computationally, we plan to explicitly model antigen dynamics during affinity maturation (as we did in ref. 59) and to study temporal evolution of antigen and information extraction. This will confirm whether optimizing affinity discrimination measured by Fisher information would indeed maximize the adaptation rate of GC B cell populations. We will also study if population dynamics of T helper cells that develop in concert with GC B cells (60) may further promote the adaptive potential of the B cell ensemble.

Simplifying assumptions made in this work point to future directions. We assumed that contact patterns are preformed and that force acting on bound complexes has no dependence on mechanics or shape of the membrane; conversely, active stresses do not alter the contact pattern. To account for these feedback mechanisms, one approach is to formulate a continuum theory describing spatiotemporal evolution of the contact pattern and the membrane profile, driven by coupled membrane mechanics, binding kinetics, and motor activity. Such a theory should yield rich dynamics of information acquisition, revealing functional constraints such as speed-accuracy tradeoffs ubiquitous in sensory systems. Another interesting extension is to consider alternative force dependencies of reaction rates. Immune B cells and T cells are known to display distinct force-lifetime characteristics: T cells exhibit catch bonds in which receptor–ligand binding is strengthened under applied force (61), whereas B cells primarily use slip bonds that destabilize as force stretches the bond (38). We will examine how pattern formation and information extraction depend on force responses of receptor–ligand bonds, to shed light on why the two arms of adaptive immunity deploy distinct cellular contact patterns and force-lifetime characteristics for antigen recognition.

## Materials and Methods

*SI Appendix* contains a detailed description of the physical-information framework that comprises the physical model of tug-of-war antigen extraction, the governing equations for the distributions of affinity readouts (cluster lifetime and extraction level), and the formulation for evaluating the amount of Fisher information (discriminatory power) these readouts contain. Moment equations are derived and the resulting first two moments allow to estimate an information lower bound. We further establish an upper bound of selection fidelity (ranking fidelity among clones with similar affinities) in terms of acquired information and derive an expression for the optimal cluster size that maximizes total information as a function of the affinity gap between receptor–antigen and antigen–tether interactions. We finally present analytical and numerical results of the effect of rebinding on information extraction and compare the Bell’s model and landscape models of bond rupture.

## Supplementary Material

Appendix 01 (PDF)

## Data Availability

All study data are included in the article and/or *SI Appendix*.
