# Supplementary material for: Physical extraction of antigen and information
Source: Proc Natl Acad Sci U S A. 2024 Sep 20;121(39):e2320537121. doi: 10.1073/pnas.2320537121 (PMC11441497; doi:10.1073/pnas.2320537121)
Supplement: Supplementary file 1 — Appendix 01 (PDF) [file pnas.2320537121.sapp.pdf]

Supplementary Information: Physical extraction of antigen and information

Hongda Jiang and Shenshen Wang
Department of Physics and Astronomy, University of California, Los Angeles, Los Angeles, CA 90095, USA

CONTENTS

I. Tug-of-war model of antigen extraction 1

II. Readout distribution 2
A. Cluster lifetime distribution and its moments 2
B. Extraction level distribution and its moments 4
C. Ensemble of dissociation trajectories 5

III. Evaluation of Fisher information 5
A. Constant force per bond 6
1. FI in cluster lifetime 6
2. FI in extraction level 8
3. FI in dissociation trajectories 8
B. Load sharing with negligible rebinding 9
1. FI in cluster lifetime 9
2. FI in extraction level 9
3. FI in dissociation trajectories 10
C. Load sharing with finite rebinding 10

IV. Relationship between Fisher information and selection fidelity 10
A. Formalism: Fisher information sets an upper bound for selection fidelity 10
B. Examples: validity of the upper bound 12
C. Generalization to top-K ranking fidelity 12

V. Optimal cluster size 13

VI. Influence of rebinding on information extraction 14

VII. Bell's phenomenological model vs landscape model of bond rupture 14

References 22

I. TUG-OF-WAR MODEL OF ANTIGEN EXTRACTION

We earlier developed a coarse-grained model of tug-of-war antigen extraction[1], in which BCR-Ag-tether 3-body complexes connect a B cell to the APC and stochastically rupture under pulling stress exerted by B cell cortical contraction upon productive BCR-Ag binding. In a mean-field description of independent complexes (without rebinding), we formulated antigen extraction as a two-dimensional first passage problem, because estimating the probability of successful extraction is equivalent to comparing the first passage times to exceeding each of the bond rupture lengths. Under modest constant pulling forces, bond lifetime distributions are nearly exponential and the extraction probability is simply $\eta = 1/(1 + \tau_a/\tau_b)$, where $\tau_b = 1/k_b$ and $\tau_a = 1/k_a$ are respectively the mean lifetimes (i.e. inverse off-rates) of the tugging (BCR-Ag) and tethering (Ag-tether) interactions. This simple expression quantifies the intuition that the essence of tug-of-war signal extraction is competitive rupture of coupled molecular bonds in the pulling direction.

Now, dissociation of a cluster of N 3-body complexes requires simultaneous rupture of all complexes. With rebinding, a broken bond can reform as long as some complexes remain to hold the cell membranes in close proximity. To describe tug-of-war antigen extraction via a receptor cluster, we consider both unbinding/rebinding of the BCR-Ag bonds and dissociation/association of the Ag-tether bonds; the single-bond off-rates k_a and k_b depend on intrinsic

bond properties, i.e., free energy barriers (E_a, E_b) and bond extensions (x_a, x_b) corresponding to thermally-driven bond rupture without pulling force, as well as tugging force per bond f (Eq. 2 in the main text).

The state of the system evolves as a result of stochastic events of bond formation and dissociation that occur one at a time. Thus, the probability, $P_{m,n}(t)$, of having m 3-body complexes and n BCR-bound antigens at time t, follows a one-step master equation (Eq. 1 in the main text):

$$\frac{\mathrm{d}P_{m,n}(t)}{\mathrm{d}t} = W_{m,n}P_{m,n}(t). \tag{S1}$$

Here $\{W_{m,n}\}$ denotes the matrix-valued evolution operator that has no explicit time dependence. Specifically,

$$W_{m,n} = (\xi^{1,-1} - 1)mk_{a,m} + (\xi^{1,0} - 1)mk_{b,m} + (\xi^{-1,0} - 1)(N - m - n)k_{\mathrm{on}} + (\xi^{-1,1} - 1)nk_{\mathrm{on}}.$$

The step operator $\xi^{i,j}$ acts on the function that follows it according to $\xi^{i,j}f_{m,n} = f_{m+i,n+j}$. The first two terms describe dissociation of a closed bond on the Ag-tether side and on the BCR-Ag side, respectively, within a cluster of size m. The other two terms account for the formation of a new bond on either side of an antigen in the presence of m 3-body complexes and n BCR-bound antigens. Note that the single-bond off-rates $k_{a,m}$ and $k_{b,m}$ may depend on cluster size m (e.g. in the load-sharing case). We assume an identical on-rate k_{on} for any single-bond association event for simplicity. Starting with maximum bond formation (all N antigens being present in the form of 3-body complexes at $t = 0$), the cluster size fluctuates over time until all complexes break; the cell locally detaches and an extraction attempt ends. Depending on whether and how force applies, distinct distributions of an affinity readout result.

II. READOUT DISTRIBUTION

Evaluation of Fisher information requires a readout distribution as the input. In this section, we calculate the distribution of two readouts of receptor quality – cluster lifetime and extraction level, both resulting from the physical process of antigen extraction through pulling on receptor clusters. Both distributions are obtained at the time of cluster dissociation. We evaluate their mean and variance (analytically or numerically) and determine the ensemble of dissociation trajectories in special cases. These results will be used to calculate Fisher information (or its lower bound) in the next section.

A. Cluster lifetime distribution and its moments

Cluster lifetime is defined as the time from maximum bond formation $(m = N)$ at $t = 0$ to complete dissociation of the cluster; an absorbing boundary condition at $m = 0$ prevents re-association of a dissociated cluster. Hence, the cluster lifetime distribution is a first-passage time (FPT) distribution

$$P_\tau(t) = -\frac{\mathrm{d}}{\mathrm{d}t}S(t), \tag{S2}$$

where the survival probability, $S(t) = \sum_{m=1}^{N} P_m(t)$, is the likelihood that by time t the cluster size remains finite.

The probability distribution of cluster size, $P_m(t) = \sum_{n=0}^{N-m} P_{m,n}(t)$, evolves over time according to a one-step birth-death process described by the following forward master equation:

$$\frac{\mathrm{d}P_m(t)}{\mathrm{d}t} = \mathcal{A}_m^{(f)}P_m(t) = [(\xi^1 - 1)r_m + (\xi^{-1} - 1)g_m]P_m(t), \tag{S3}$$

where $\mathcal{A}_m^{(f)}$ is the forward evolution operator. The forward (association) and reverse (dissociation) rates are respectively given by

$$g_m = (N - m)k_{\mathrm{on}}, \quad r_m = mk_0\left[e^{-\beta(E_a - f(m)x_a)} + e^{-\beta(E_b - f(m)x_b)}\right]. \tag{S4}$$

Here $f(m)$ denotes the force magnitude per bond; it can be a constant, i.e. $f(m) = f$, if the total force scales linearly with the cluster size, whereas in the case of loading sharing, $f(m) = F/m$, that is, a constant total force F is evenly shared among m remaining 3-body complexes. The absorbing boundary at cluster dissociation is imposed by setting $g_0 = 0$.

Alternatively, the FPT distribution can be obtained from solving the equivalent backward equation,

$$\frac{\mathrm{d}P_\tau(t|m_0)}{\mathrm{d}t} = \mathcal{A}^{(b)}_{m_0} P_\tau(t|m_0) = [r_{m_0}(\xi^{-1}-1) + g_{m_0}(\xi^{1}-1)]P_\tau(t|m_0), \tag{S5}$$

where $P_\tau(t|m_0)$ is the FPT distribution starting from the state $m = m_0$ at $t = 0$. $\mathcal{A}^{(b)}_m$ is the backward operator acting on m_0, i.e., $\xi^{\pm 1}P_\tau(t|m_0) = P_\tau(t|m_0 \pm 1)$. Note that $P_\tau(t|0) = \delta(t)$ which vanishes for any $t > 0$, representing the absorbing boundary condition. That is, a dissociated cluster is not allowed to reattach. We are interested in finding $P_\tau(t|N)$, the FPT distribution starting from the fully bound state, $m_0 = N$, in which all antigens are bound in 3-body complexes. While the backward equation is not necessarily easier to solve than the forward equation if one aims for the full FPT distribution, it provides a convenient way for calculating the moments of the FPT iteratively, due to the fact that the backward evolution operator acts on the initial state.

Define the q-th moment of the FPT starting from cluster size m_0 as follows

$$\tau^{(q)}_{m_0} \equiv \int_0^\infty t^q P_\tau(t|m_0)dt. \tag{S6}$$

Applying the backward operator on both sides, we obtain a recursive relation

$$\mathcal{A}^{(b)}_{m_0}\tau^{(q)}_{m_0} = \int_0^\infty t^q \frac{dP_\tau(t|m_0)}{dt}dt = -q\tau^{(q-1)}_{m_0}, \tag{S7}$$

where we have used the boundary condition $P_\tau(t=\infty|m_0) = 0$. Thus, to determine the moment of each order, we need to solve N coupled algebraic equations as m_0 runs from 1 to N.

Explicitly, Eq. S7 reads

$$r_{m_0}\tau^{(q)}_{m_0-1} + g_{m_0}\tau^{(q)}_{m_0+1} - (r_{m_0}+g_{m_0})\tau^{(q)}_{m_0} = -q\tau^{(q-1)}_{m_0}.$$

We rearrange terms and get

$$\tau^{(q)}_{m_0} - \tau^{(q)}_{m_0-1} = \frac{q\tau^{(q-1)}_{m_0}}{r_{m_0}} + \frac{g_{m_0}}{r_{m_0}}(\tau^{(q)}_{m_0+1} - \tau^{(q)}_{m_0}).$$

Solving this set of equations iteratively with boundary conditions $\tau^{(q)}_0 = 0$ and $\tau^{(q)}_N - \tau^{(q)}_{N-1} = q\tau^{(q-1)}_N/r_N$, we obtain

$$\tau^{(q)}_{m_0} - \tau^{(q)}_{m_0-1} = \frac{q\tau^{(q-1)}_{m_0}}{r_{m_0}} + \sum_{j=m_0+1}^{N} q\tau^{(q-1)}_j \frac{\prod_{k=m_0}^{j-1} g_k}{\prod_{k=m_0}^{j} r_k}.$$

We can thus establish a relationship between $\tau^{(q)}_{m_0}$ and $\tau^{(q-1)}_i$ (with i running from 1 to N) to get the following expression of the q-th moment

$$\tau^{(q)}_{m_0} = \sum_{i=1}^{m_0} \frac{q\tau^{(q-1)}_i}{r_i} + \sum_{i=1}^{m_0}\sum_{j=i+1}^{N} q\tau^{(q-1)}_j \frac{\prod_{k=i}^{j-1} g_k}{\prod_{k=i}^{j} r_k}. \tag{S8}$$

Specifically, the mean cluster lifetime (i.e. the first moment) reads

$$\mu_\tau = \tau^{(1)}_N = \sum_{i=1}^{N} \frac{1}{r_i} + \sum_{i=1}^{N-1}\sum_{j=i+1}^{N} \frac{\prod_{k=i}^{j-1} g_k}{\prod_{k=i}^{j} r_k}. \tag{S9}$$

The first term is simply the mean lifetime without rebinding ($k_{\mathrm{on}} = 0$), and the second term describes how rebinding significantly prolongs cluster lifetime, with a leading order term of k_{on}^{N-1} in the limit of fast rebinding. Note that $g_N = 0$.

Similarly, the second moment follows from Eq. S8 as follows

$$\tau^{(2)}_N = \sum_{i=1}^{N} \frac{2\tau^{(1)}_i}{r_i} + \sum_{i=1}^{N-1}\sum_{j=i+1}^{N} 2\tau^{(1)}_j \frac{\prod_{k=i}^{j-1} g_k}{\prod_{k=i}^{j} r_k}. \tag{S10}$$

The variance is thus given by

$$\sigma^2_\tau = \tau^{(2)}_N - (\tau^{(1)}_N)^2. \tag{S11}$$

Even when the analytical expression of the FPT distribution is not available, one can use Eq. S9 to Eq. S11 to obtain the first two moments which would suffice to estimate the lower bound of Fisher information (Eq. 9 in the main text).

B. Extraction level distribution and its moments

The distribution of antigen extraction level is the asymptotic solution of Eq. S1 at the absorbing boundary ($m = 0$):

$$P_{n_{\rm Ag}}(n) \equiv P_{0,n}(t = \infty). \tag{S12}$$

For a general reaction matrix, $W_{m,n}$, as we show below, instead of solving the differential equations, one can construct a set of linear algebraic equations and compute the asymptotic distribution.

Taking advantage of the fact that the evolution operator has no explicit time dependence, we integrate the master equation over time and obtain

$$P_{m,n}(\infty) - P_{m,n}(0) = \int_0^\infty \dot{P}_{m,n}(t)dt = W_{m,n}\int_0^\infty P_{m,n}(t)dt. \tag{S13}$$

This equation says that the change in occupation probability of a state is due to a net probability current flowing into it from the adjacent states. In particular, for the target state at the absorbing boundary, the asymptotic distribution is a sum of two time-integrated distributions:

$$P_{0,n}(\infty) = k_{a,1}\int_0^\infty P_{1,n-1}(t)dt + k_{b,1}\int_0^\infty P_{1,n}(t)dt. \tag{S14}$$

This observation motivates us to define a time-integrated probability for each non-absorbing state ($m > 0$), that is,

$$Q_{m,n} \equiv \int_0^\infty P_{m,n}(t)dt. \tag{S15}$$

Given the initial condition $P_{m,n}(0) = \delta_{m,N}\delta_{n,0}$ and that the asymptotic distribution at any non-absorbing state obeys $P_{m,n}(\infty) = 0$, Eq. S13 becomes a linear set of equations for the integrated probabilities $\{Q_{m,n}\}$:

$$\begin{aligned} -\delta_{m,N}\delta_{n,0} = {} & (m+1)k_{a,m+1}Q_{m+1,n-1} + (m+1)k_{b,m+1}Q_{m+1,n} + (n+1)k_{\rm on}Q_{m-1,n+1} + \\ & (N-m-n+1)k_{\rm on}Q_{m-1,n} - [mk_{a,m} + mk_{b,m} + (N-m)k_{\rm on}]Q_{m,n}, \end{aligned} \tag{S16}$$

where $1 \le m \le N$ and $0 \le n \le N - m$. Note that $Q_{0,n} = 0$. Therefore, there are in total $N(N + 1)/2$ coupled linear equations to determine the same number of unknowns, $\{Q_{m,n}\}$. By solving them, we can find the extraction level distribution through

$$P_{n_{\rm Ag}}(n) = k_{a,1}Q_{1,n-1} + k_{b,1}Q_{1,n}. \tag{S17}$$

In this way, we convert the original $N^2/2$ *ordinary differential equations* in Eq. S1 into $N^2/2$ *linear algebraic equations* in Eq. S16 that can be solved at a much lower computational cost.

Furthermore, if we only need to determine the first and second moments, the task reduces further to solving $3N$ linear equations that we derive below. Define the order-k "time-integrated moments" of extraction level n:

$$R_m^{(k)} \equiv \sum_{n=0}^{N} n^k Q_{m,n}. \tag{S18}$$

Note that $Q_{m,n} = 0$ if $m + n > N$. We then have

$$\begin{aligned} \langle n_{\rm Ag}\rangle \equiv \sum_{n=1}^{N} nP_{n_{\rm Ag}}(n) &= k_{a,1}\sum_{n=1}^{N} nQ_{1,n-1} + k_{b,1}\sum_{n=1}^{N} nQ_{1,n} \\ &= k_{a,1}\left(R_1^{(1)} + R_1^{(0)}\right) + k_{b,1}R_1^{(1)}. \end{aligned} \tag{S19}$$

We used $\sum_{n=0}^{N} nQ_{1,n-1} = \sum_{n=0}^{N-1}(n+1)Q_{1,n} = \sum_{n=0}^{N}(n+1)Q_{1,n}$. Similarly,

$$\begin{aligned} \langle n_{\rm Ag}^2\rangle \equiv \sum_{n=1}^{N} n^2P_{n_{\rm Ag}}(n) &= k_{a,1}\sum_{n=1}^{N} n^2Q_{1,n-1} + k_{b,1}\sum_{n=1}^{N} n^2Q_{1,n} \\ &= k_{a,1}\left(R_1^{(2)} + 2R_1^{(1)} + R_1^{(0)}\right) + k_{b,1}R_1^{(2)}. \end{aligned} \tag{S20}$$

Thus, $\langle n_{\rm Ag}\rangle$ and $\langle n_{\rm Ag}^2\rangle$ can be calculated once we know $R_1^{(0)}, R_1^{(1)}$ and $R_1^{(2)}$. It is straightforward to construct linear equations for $R_m^{(k)}$ based on Eq. S16.

For $k=0$, we simply sum over n on both sides of Eq. S16 and obtain

$$\begin{aligned}-\delta_{mN} =&(m+1)(k_{a,m+1}+k_{b,m+1})R_{m+1}^{(0)} + (N-m+1)k_{\rm on}R_{m-1}^{(0)}\\ &-(mk_{a,m}+mk_{b,m}+(N-m)k_{\rm on})R_m^{(0)}, \qquad m=1,2,...,N.\end{aligned} \tag{S21}$$

In addition, we know $R_0^{(0)}=0$ and get $R_1^{(0)}=1/(k_{a,1}+k_{b,1})$ from Eq. S17 based on $\sum_n P_{n_{\rm Ag}}(n)=1$. We can thus calculate $R_m^{(0)}$ iteratively using Eq. S21.

For $k=1$, we have

$$\begin{aligned}0 =&(m+1)k_{a,m+1}(R_{m+1}^{(1)}+R_{m+1}^{(0)}) + (m+1)k_{b,m+1}R_{m+1}^{(1)}\\ &+(N-m)k_{\rm on}R_{m-1}^{(1)} - (mk_{a,m}+mk_{b,m}+(N-m)k_{\rm on})R_m^{(1)}, \qquad m=1,2,...,N.\end{aligned} \tag{S22}$$

Using $R_0^{(1)}=0$ and $R_{N+1}^{(1)}=0$ and the calculated $R_m^{(0)}$, one can find $R_m^{(1)}$.

For $k=2$, we obtain

$$\begin{aligned}0 =&(m+1)k_{a,m+1}(R_{m+1}^{(2)}+2R_{m+1}^{(1)}+R_{m+1}^{(0)}) + (m+1)k_{b,m+1}R_{m+1}^{(2)}\\ &+k_{\rm on}[(N-m-1)R_{m-1}^{(2)}+R_{m-1}^{(1)}] - (mk_{a,m}+mk_{b,m}+(N-m)k_{\rm on})R_m^{(2)}, \quad m=1,2,...,N.\end{aligned} \tag{S23}$$

Here, $R_m^{(2)}$ can be found using $R_m^{(0)}$ and $R_m^{(1)}$ determined above. Therefore, by solving $3N$ linear equations (Eq. S21 to Eq. S23), we can find the first two moments of extraction level, which allow to estimate the lower bound of Fisher information.

C. Ensemble of dissociation trajectories

In a particular realization of the extraction process defined by Eq. S1, the sequence of reaction events constitutes a dissociation trajectory. Within our model, we denote a trajectory by a pair of vectors $(\mathbf{t},\mathbf{s})=\{(t_i,s_i); i=1,...,N_R\}$, where t_i represents the waiting time between reaction $(i-1)$ and reaction i, whereas s_i labels the type of reaction i. N_R is the total number of reactions leading toward complete dissociation. We want to write down the probability distribution (i.e. statistical ensemble) of such trajectories, $P(\mathbf{t},\mathbf{s})$.

For simplicity and physical clarity, we demonstrate the case with negligible rebinding. In this scenario, only two types of reactions can occur: rupture of the APC-Ag bond (denoted as $s_i=s_a$) and breaking of the BCR-Ag bond (denoted as $s_i=s_b$). The total number of reactions is simply the total number of antigens, i.e., $N_R=N$. It thus follows

$$\begin{aligned}P(\mathbf{t},\mathbf{s}) &= P(\mathbf{t})\times P(\mathbf{s}),\\ P(\mathbf{t}) &= \prod_{i=1}^{N} m(i)\left(k_{a,m(i)}+k_{b,m(i)}\right)e^{-m(i)(k_{a,m(i)}+k_{b,m(i)})t_i},\\ P(\mathbf{s}) &= \prod_{i=1}^{N}\left[\frac{k_{a,m(i)}}{k_{a,m(i)}+k_{b,m(i)}}\delta_{s_i,s_a}+\frac{k_{b,m(i)}}{k_{a,m(i)}+k_{b,m(i)}}\delta_{s_i,s_b}\right].\end{aligned} \tag{S24}$$

Here $m(i)=N-i+1$ is the number of remaining 3-body complexes *prior to* reaction i. Here, the waiting time $\mathbf{t}$ and reaction type $\mathbf{s}$ are independent random variables: $\{t_i\}$ follow an exponential distribution with mean waiting times $[m(i)\left(k_{a,m(i)}+k_{b,m(i)}\right)]^{-1}$, whereas $\{s_i\}$ follow a Bernoulli distribution. The formulation of Eq. S24 is based on the fundamental premise of stochastic chemical kinetics, which assumes that the probability that a reaction of type s_i occurs after a sojourn time of length t_i is given by $P(t_i,s_i|m(i))=a_{s_i}(m(i))e^{-t_i\sum_{s_j}a_{s_j}(m(i)))}$, where $a_{s_i}(m(i))$ denotes the propensity of reaction i given the current state $m(i)$.

III. EVALUATION OF FISHER INFORMATION

In this section, we use Fisher information to evaluate distinguishability of receptor quality based on alternative readouts. Fisher information can take several equivalent forms with different emphases. For one, the information

content is determined by the mean square gradient of the log-likelihood function in affinity space:

$$\mathcal{I} = \langle \left(\frac{\partial \ln P(\mathbf{y}; E_b)}{\partial E_b} \right)^2 \rangle = \int \left(\frac{\partial \ln P(\mathbf{y}; E_b)}{\partial E_b} \right)^2 P(\mathbf{y}; E_b) d\mathbf{y}. \tag{S25}$$

For another, distinguishability is set by the mean negative curvature of the log likelihood. Intuitively, a stronger curvature indicates a higher sensitivity of the corresponding readout Y to changes in affinity E_b, and hence a greater amount of information contained in Y about E_b:

$$\mathcal{I} = - \int \frac{\partial^2 \ln P(\mathbf{y}; E_b)}{\partial E_b^2} P(\mathbf{y}; E_b) d\mathbf{y}. \tag{S26}$$

Below we present the results for biologically plausible scenarios of extraction setup with increasing complexity: (1) constant force per bond (independent complexes); (2) load sharing with negligible rebinding; (3) load sharing with finite rebinding. We can obtain analytical understanding for the first two cases, based on a full readout distribution or its moments, and for the last we resort to numerical evaluation.

A. Constant force per bond

When complexes are decoupled and rupture events independent, we have $f(m) = f$. In this case, off rates denoted by $k_{a,m} = k_a$ and $k_{b,m} = k_b$ are constant, irrespective of the cluster state.

1. *FI in cluster lifetime*

In general, the lower bound of FI can be calculated from the mean μ_τ and variance σ_τ^2 of the cluster lifetime distribution,

$$\tilde{\mathcal{I}}_\tau = \frac{1}{\sigma_\tau^2} \left(\frac{\mathrm{d}\mu_\tau}{\mathrm{d}E_b} \right)^2. \tag{S27}$$

To acquire analytical understanding, we consider two limits: negligible rebinding ($k_{\text{on}} = 0$) and fast rebinding ($k_{\text{on}} \gg k_a, k_b$).

a. negligible rebinding Without rebinding ($k_{\text{on}} = 0$), cluster lifetime is set by the unbinding time of the longest lasting bond and thus follows an extreme value distribution. Since individual bond lifetimes are independent identically distributed random variables, which follow an exponential distribution with mean $(k_a + k_b)^{-1}$, the distribution of cluster lifetime $P_\tau(t)$ is of Gumble type. Denoting the off rate of a 3-body complex with $\lambda = k_a + k_b$, one can solve Eq. S5 (backward master equation) with $g_m = 0$ and $r_m = m\lambda$ to obtain

$$P_\tau(t) = N\lambda e^{-\lambda t}(1 - e^{-\lambda t})^{N-1}. \tag{S28}$$

This self-evident expression represents the probability that, among N identical bonds, the longest-lived one lasts until time t while all others break sooner. The cumulative distribution function (CDF), $\int_0^t P_\tau(t')dt' = (1 - e^{-\lambda t})^N$, has an intuitive meaning as the probability that none of the complexes survives longer than time t.

The first two moments follow readily from either Eq. S28 or from Eq. S9 to Eq. S11:

$$\mu_\tau = \sum_{i=1}^{N} \frac{1}{i\lambda} = \frac{H_N}{\lambda}, \qquad \sigma_\tau^2 = \sum_{i=1}^{N} \frac{1}{(i\lambda)^2} = \frac{\pi^2}{6\lambda^2} - \frac{\psi^{(1)}(N+1)}{\lambda^2}. \tag{S29}$$

Here $H_N = \sum_{i=1}^{N} 1/i$ is the Harmonic number, whereas $\psi^{(1)}(N+1) = \sum_{i=N+1}^{\infty} 1/i^2$. In the limit of large N, we have

$$\mu_\tau \approx \frac{\ln N}{\lambda}, \qquad \sigma_\tau^2 \approx \frac{\pi^2}{6\lambda^2}. \tag{S30}$$

Note that the mean lifetime increases logarithmically with the initial cluster size N while the variance remains nearly constant, which is characteristic of the Gumbel distribution.

To calculate FI in cluster lifetime, we directly evaluate Eq. S26. Explicitly, the curvature of the log likelihood reads

$$\frac{\partial^2 \ln P_\tau(t)}{\partial E_b^2} = -\beta^2 k_b^2 \left[\frac{1}{\lambda^2} + (N-1)\frac{t^2 e^{-\lambda t}}{(1-e^{-\lambda t})^2}\right] + \beta^2 k_b \left[\frac{1}{\lambda} - t + (N-1)\frac{t e^{-\lambda t}}{1-e^{-\lambda t}}\right]. \tag{S31}$$

Here we used $\frac{\partial}{\partial E_b} = -\beta k_b \frac{\partial}{\partial k_b}$. For $N > 2$, averaging over $P_\tau(t)$ yields

$$\mathcal{I}_\tau = \frac{\beta^2 k_b^2}{\lambda^2}\Big[1 + \frac{N}{N-2}\Big(\frac{1}{(N-1)^2} + (\gamma-2)\gamma + \frac{\pi^2}{6} + \psi^{(0)}(N)\left[2(\gamma-1) + \psi^{(0)}(N)\right] - \psi^{(1)}(N-1)\Big)\Big]. \tag{S32}$$

Here γ is the Euler constant and $\psi^{(n)}(z)$ is the Polygamma function of order n.

For large N, $\psi^{(0)}(N) \approx \ln(N) + \mathcal{O}(1/N)$ and $\psi^{(1)}(N) = 1/N + \mathcal{O}(1/N^2)$. Eq. S32 thus simplifies and becomes

$$\mathcal{I}_\tau \approx \beta^2 \frac{(\ln N)^2}{(1+e^{\beta\Delta E})^2}, \tag{S33}$$

where $\Delta E = E_b - E_a - f(x_b - x_a)$. $\mathcal{I}_\tau$ has a weak dependence on the initial cluster size N because the information about the statistics of waiting times between successive rupture events is lost when only the total duration is measured.

b. fast rebinding In this limit, $k_{\text{on}} \gg k_a, k_b$, the FPT distribution appears to follow an exponential distribution, as depicted in Fig. S3B. This is because frequent rebinding creates an effective barrier against cluster dissociation; noise-driven barrier crossing becomes exponentially rare. To provide the mathematical basis of this behavior, it is informative to look at how rebinding influences the mean and variance of the cluster lifetime.

Given constant k_a and k_b, the products in Eq. S9 to Eq. S11 become Binomial coefficients. Letting $\gamma = k_{\text{on}}/\lambda$, we get

$$\mu_\tau = \frac{1}{\lambda}\sum_{i=1}^{N}\sum_{j=i}^{N}\frac{\binom{N}{j}}{\binom{N}{i}}\frac{\gamma^{j-i}}{i} = \frac{1}{\lambda}\sum_{i=1}^{N}\frac{(1+\gamma)^{i-1}}{i}. \tag{S34}$$

The second equality can be proven by carrying out the binomial expansion and collecting terms in different orders of γ. Since μ_τ is a polynomial in γ up to order $N-1$, for $\gamma \gg 1$, leading order terms take over, and the increase in μ_τ with k_{on} becomes exponential. Similarly, we obtain the standard deviation which is also a polynomial in γ up to order $N-1$,

$$\sigma_\tau = \frac{1/\lambda}{1+\gamma}\sqrt{\left[\sum_{i=1}^{N}\binom{N}{i}\frac{\gamma^i}{i}\right]^2 + \sum_{i=1}^{N}\left[\binom{N}{i}\frac{2\gamma^i}{i^2} + \frac{1}{i^2}\right]}.$$

In the limit of fast rebinding ($\gamma \gg 1$), the mean and standard deviation become identical – a feature of exponential distribution; they both exhibit a polynomial in γ of order $N-1$:

$$\sigma_\tau \approx \mu_\tau \approx \frac{1}{\lambda}\frac{\gamma^{N-1}}{N}. \tag{S35}$$

This suggests that, compared to increasing the cluster size, frequent rebinding is far more effective at prolonging the cluster lifetime; cluster dissociation is exponentially suppressed:

$$P_\tau(t) \approx \frac{1}{\mu_\tau}e^{-t/\mu_\tau}. \tag{S36}$$

Direct evaluation of Eq. S25 then yields the information content

$$\mathcal{I}_{\tau,k_{\text{on}}\gg\lambda} = \beta^2\Big(\frac{N}{1+e^{\beta\Delta E}}\Big)^2. \tag{S37}$$

Note that fast rebinding results in a much stronger scaling with cluster size, $\mathcal{I}_\tau \sim N^2$, than that in the absence of rebinding, $\mathcal{I}_\tau \sim (\ln N)^2$. However, the scaling with effective BCR affinity remains unchanged: at high affinities, $\mathcal{I}_\tau \sim e^{-2\beta\Delta E}$.

2. FI in extraction level

a. $k_{\rm on} = 0$ Without rebinding, Eq. S17 becomes the recursive relation of Binomial coefficients: $\binom{N-m}{n} = \binom{N-m-1}{n} + \binom{N-m-1}{n-1}$. That is, the level of antigen extraction assumes a Binomial distribution

$$P_{n_{\rm Ag}}(n) = \binom{N}{n} \frac{k_a^n k_b^{N-n}}{(k_a+k_b)^N}. \tag{S38}$$

This agrees with the intuition that antigen extraction, without coupling or rebinding, is a Bernoulli process, because the probability of acquiring antigen is the same at each extraction attempt.

b. $k_{\rm on} > 0$ Even if rebinding is considerable, when two binding interfaces have the same on-rate, whether extraction would occur or not depends only on the last unbinding event. In other words, intermediate steps of unbinding and rebinding do not alter the chance of extraction, as long as the magnitude of pulling force acting on each complex remains constant. Hence, $n_{\rm Ag}$ follows the same Binomial distribution as above. One can confirm it by substituting Eq. S38 into Eq. S17 with non-vanishing $k_{\rm on}$.

Therefore, under constant force per bond, Fisher information in extraction level reads

$$\mathcal{I}_{n_{\rm Ag}} = -\sum_{n=1}^{N} P(n) \frac{\partial^2 \ln P(n)}{\partial E_b^2} = \beta^2 \sum_{n=1}^{N} P(n) N \frac{k_a k_b}{(k_a+k_b)^2} = \beta^2 N \frac{e^{\beta\Delta E}}{(1+e^{\beta\Delta E})^2} \tag{S39}$$

Different from $\mathcal{I}_\tau$, $\mathcal{I}_{n_{\rm Ag}}$ depends linearly on N and falls more slowly with increasing affinity as $\mathcal{I}_{n_{\rm Ag}} \sim e^{-\beta\Delta E}$.

3. FI in dissociation trajectories

We can calculate analytically the information encoded in extraction trajectories, if complexes are decoupled ($k_{a,m} = k_a$ and $k_{b,m} = k_b$) and rebinding is negligible ($k_{\rm on} = 0$). In this case, Eq. S24 simplifies and we directly evaluate Eq. S26 as follows. First, the log likelihood is given by

$$\begin{aligned} \ln P(\{t_i, s_i\}) &= \sum_{i=1}^{N} \ln\left[m(i)(k_a+k_b)\right] - m(i)(k_a+k_b)t_i \\ &+ \sum_{i=1}^{N} \ln\left[\frac{k_a}{k_a+k_b}\delta_{s_i,s_a} + \frac{k_b}{k_a+k_b}\delta_{s_i,s_a}\right] \end{aligned} \tag{S40}$$

where $m(i) = N - i + 1$ is the number of remaining 3-body complexes prior to reaction i. Using $\partial/\partial E_b = -\beta k_b \partial/\partial k_b$, we obtain

$$-\frac{\partial^2 \ln P(\{t_i, s_i\})}{\partial E_b^2} = \beta^2 \sum_{i=1}^{N} m(i) t_i k_b$$

Hence,

$$\mathcal{I}_{\rm full} = \langle -\frac{\partial^2 \ln P(\{t_i, s_i\})}{\partial E_b^2} \rangle = \beta^2 \frac{N k_b}{k_a+k_b} = \beta^2 \frac{N}{1+e^{\beta\Delta E}} \tag{S41}$$

Note that full Fisher information contained in the dissociation trajectories is equal to the expected number of bond rupture events that occur on the BCR-Ag side during the extraction process, that is, $\langle N - n_{\rm Ag}\rangle = N k_b/(k_a+k_b)$. This is expected because each rupture event provides an independent measurement of BCR-Ag affinity.

In addition, we found that

$$\mathcal{I}_{\{t_i\}} = \beta^2 N \frac{1}{(1+e^{\beta\Delta E})^2} > \mathcal{I}_\tau, \qquad \mathcal{I}_{\{s_i\}} = \beta^2 N \frac{e^{\beta\Delta E}}{(1+e^{\beta\Delta E})^2} = \mathcal{I}_n$$

This suggests that while measurements of cluster lifetime lose information contained in the distribution of waiting times, the level of antigen extraction preserves information encoded in the sequence of reaction types and thus approaches full information obtainable from extraction trajectories at high affinities.

B. Load sharing with negligible rebinding

When a fixed load distributes evenly across a cluster, single-bond off rates now depend on the system state, because force acting on each remaining bond depends on the current cluster size (i.e. the number of persistent bonds that share the load). In general, this effective coupling between bonds may yield complicated expressions of the readout distribution that make analytical calculation challenging. We instead seek the lower bound $\tilde{\mathcal{I}}$ by evaluating the first two moments of the readout distribution. This approximation matches extremely well with the exact results from solving the master equation, even for modest cluster sizes.

1. *FI in cluster lifetime*

According to Eq. S9 and Eq. S11, we have

$$\begin{aligned} \mu_\tau &= \sum_{i=1}^{n} \langle t_i \rangle = \sum_{i=1}^{N} \frac{1}{r_i}, \\ \sigma_\tau^2 &= \sum_{i=1}^{N} \sigma_{t_i}^2 = \sum_{i=1}^{N} \frac{1}{r_i^2} \end{aligned} \tag{S42}$$

where $r_i = i(k_{a,i} + k_{b,i}) = ik_0 \left[e^{-\beta(E_a - Fx_a/i)} + e^{-\beta(E_b - Fx_b/i)}\right]$. Thus,

$$\tilde{\mathcal{I}}_\tau \simeq \beta^2 \frac{1}{\sum\limits_{i=1}^{N} r_i^{-2}} \left(\sum_{i=1}^{N} \frac{ik_{b,i}}{r_i^2} \right)^2 \tag{S43}$$

where we used $\partial r_i / \partial E_b = -i\beta k_{b,i}$.

2. *FI in extraction level*

The mean and variance of extraction level are respectively

$$\begin{aligned} \mu_n &= \sum_{i=1}^{N} \eta_i = \sum_{i=1}^{N} \frac{k_{a,i}}{k_{a,i} + k_{b,i}}, \\ \sigma_n^2 &= \sum_{i=1}^{N} \eta_i (1 - \eta_i) = \sum_{i=1}^{N} \frac{k_{a,i} k_{b,i}}{(k_{a,i} + k_{b,i})^2}. \end{aligned} \tag{S44}$$

Then the lower bound of Fisher information is given by

$$\tilde{\mathcal{I}}_n = \beta^2 \sum_{i=1}^{N} \eta_i (1 - \eta_i). \tag{S45}$$

As $F \to 0$, $\tilde{\mathcal{I}}_n \to \beta^2 N e^{\beta \Delta E} / (1 + e^{\beta \Delta E})^2$, which recovers the result for independent complexes (Eq. S39).

Total information contained in the extraction level is the sum of contribution from individual extraction events:

$$\tilde{\mathcal{I}}_n = \sum_{i=1}^{N} \mathcal{I}_{i,\text{indiv.}} \tag{S46}$$

where

$$\mathcal{I}_{i,\text{indiv.}} = \beta^2 \frac{e^{\beta(\Delta E - F\Delta x/i)}}{(1 + e^{\beta(\Delta E - F\Delta x/i)})^2}$$

which peaks at $\Delta E = F\Delta x / i$.

3. FI in dissociation trajectories

Total Fisher information encoded in the extraction trajectory can be obtained from Eq. S24. First,

$$-\frac{\partial^2 \ln P(\{t_i, s_i\})}{\partial E_b^2} = \beta^2 \sum_{i=1}^{N} m(i) t_i k_{b,m(i)}$$

Therefore,

$$\begin{aligned}\mathcal{I}_{\text{full}} &= \langle -\frac{\partial^2 \ln P(\{t_i, s_i\})}{\partial E_b^2} \rangle = \beta^2 \sum_{i=1}^{N} \frac{k_{b,m(i)}}{k_{a,m(i)} + k_{b,m(i)}} \\ &= \beta^2 \sum_{i=1}^{N} \frac{1}{1 + e^{\beta(\Delta E - F\Delta x/i)}}\end{aligned} \tag{S47}$$

C. Load sharing with finite rebinding

Now that, in addition to bond coupling through load sharing, broken bonds can reform at a finite on rate, readout distributions are no longer analytically tractable. We again resort to the lower bound $\tilde{\mathcal{I}}$.

a. FI in cluster lifetime After iteratively solving for the mean μ_τ and the variance σ_τ^2 to obtain, respectively, Eq. S9 and Eq. S11, we compute the Fisher information numerically according to

$$\tilde{\mathcal{I}}_\tau = \frac{1}{\sigma_\tau^2} \Big(\frac{\mathrm{d}\mu_\tau}{\mathrm{d}E_b} \Big)^2. \tag{S48}$$

b. FI in extraction level For a small system ($N \sim 10$), we can calculate the readout distribution $P_{n_{\text{Ag}}}(n)$ by solving Eq. S16 and use the result to determine the Fisher information. For larger systems, however, we no longer have an analytical expression for the mean μ_n. We thus replace the differential form with a finite difference

$$\tilde{\mathcal{I}}_n \approx \frac{1}{\sigma_n^2} \Big(\frac{\mu_n(E_b + \epsilon) - \mu_n(E_b - \epsilon)}{2\epsilon} \Big)^2. \tag{S49}$$

In numerics, we used $\epsilon = 10^{-5} k_B T$.

IV. RELATIONSHIP BETWEEN FISHER INFORMATION AND SELECTION FIDELITY

In this section, we establish a quantitative link between Fisher information and selection fidelity. Albeit intuitive, it is satisfying to see that, through a generic scaling relationship, the acquired information sets an upper bound for selection fidelity. Since Fisher information provides a natural metric of discriminatory power, this connection quantifies the intuition that efficient selection relies on accurate discrimination.

A. Formalism: Fisher information sets an upper bound for selection fidelity

B cells estimate and rank their receptor quality through extracting antigen and competing for T cell help. Ideally, B cells that acquire, internalize, and subsequently present a larger amount of antigen (in the form of pMHCII complexes) to helper T cells will divide more, thereby expanding preferentially in the population at the expense of inferior clones. However, the dynamical process leading from BCR-Ag binding to antgien extraction is stochastic, making the affinity readouts noisy outputs. As a consequence, higher affinity cells may produce lower readouts. The fidelity of selection (affinity dependent clonal expansion) is thus limited by the accuracy of affinity ranking based on noisy readouts. Clearly, T help competition is another source of stochasticity that may further deteriorate selection fidelity. We choose to focus on the extraction stage with a goal to establish an upper bound in terms of discrimination accuracy due to physical acquisition of information. We characterize discriminatory power with Fisher information retained in a readout distribution.

Consider a population of n_B B cells with affinities E_{bi}, where $i = 1, \cdots, n_B$. With no loss of generality, we assume E_{b1} to be the highest affinity. Let $P_i(y)$ be the readout distribution associated with affinity E_{bi} (assuming that

y is continuous and $P_i(y)$ is everywhere differentiable). We define selection fidelity ξ_Y based on readout Y as the probability that a cell expressing receptors of the highest affinity will produce the largest readout value:

$$\xi_Y = \int_{-\infty}^{\infty} dy_1 P_1(y_1) \prod_{i=2}^{n_B} \int_{-\infty}^{y_1} dy_i P_i(y_i). \tag{S50}$$

This definition of selection fidelity is in fact equivalent to precision, a widely used metric for evaluating the performance of ranking algorithms.

In the regime of hard discrimination, the affinity difference between competing cells $\epsilon_i \equiv E_{b1} - E_{bi}$ is small, hence we can Taylor expand the readout distributions around $P_1(y)$,

$$P_i(y) = P_1(y) - P_1(y)s(y)\epsilon_i + \mathcal{O}(\epsilon_i^2), \tag{S51}$$

where $s(y) = \frac{\partial \ln P(y;E_b)}{\partial E_b}\big|_{E_{b1}}$ is the score function evaluated at $E_{bi} = E_{b1}$. Thus, to the linear order in ϵ_i, Eq. S50 becomes

$$\xi_Y \approx \int_{-\infty}^{\infty} dy_1 P_1(y_1) \left[\left(\prod_{i=2}^{n_B} \int_{-\infty}^{y_1} dy_i P_1(y_i) \right) - \sum_{j=2}^{n_B} \left(\prod_{i=2, i\neq j}^{n_B} \int_{-\infty}^{y_1} dy_i P_1(y_i) \right) \int_{-\infty}^{y_1} dy_j P_1(y_j) s(y_j) \epsilon_j \right].$$

We define the cumulative distribution function (CDF) of $P_1(y)$ as $G(y) = \int_{-\infty}^{y} P_1(y')dy'$ and consider separately the two terms in the expression above. The first term reads

$$\int_{-\infty}^{\infty} dy_1 P_1(y_1) [G(y_1)]^{n_B-1} = \langle G(y)^{n_B-1} \rangle = \frac{1}{n_B}.$$

Here we have used the property $\langle G(y)^\alpha \rangle = 1/(1+\alpha)$, which follows simply from integration by parts: $\int G(y)^\alpha P(y)dy = G(y)^{\alpha+1}\big|_{-\infty}^{\infty} - \alpha \int G(t)^\alpha P(y)dy = 1 - \alpha \int G(t)^\alpha P(y)dy$. The second term becomes

$$\begin{aligned}
&- \sum_{j=2}^{n_B} \epsilon_j \int_{-\infty}^{\infty} dy_1 P_1(y_1) G(y_1)^{n_B-2} \int_{-\infty}^{y_1} dy_j P_1(y_j) s(y_j) \\
&= - \sum_{j=2}^{n_B} \epsilon_j \int_{-\infty}^{\infty} dy_j P_1(y_j) s(y_j) \int_{y_j}^{\infty} dy_1 P_1(y_1) G(y_1)^{n_B-2} \\
&= \left(- \sum_{j=2}^{n_B} \epsilon_j \right) \frac{1}{n_B - 1} \int_{-\infty}^{\infty} dy P_1(y) s(y) \left(1 - G(y)^{n_B-1}\right) \\
&= \left(\sum_{j=2}^{n_B} \epsilon_j \right) \frac{1}{n_B - 1} \langle s(y) G(y)^{n_B-1} \rangle.
\end{aligned}$$

In the second line, we exchanged the order of integration between y_1 and y_j, and in the last line, we used the fact that $\langle s(y) \rangle = \int_{-\infty}^{\infty} dy P(y) s(y) = \frac{\partial}{\partial E_b} \int_{-\infty}^{\infty} dy P(y) = 0$. Hence, Eq. S50 now becomes

$$\xi_Y \approx \frac{1}{n_B} + \frac{1}{n_B - 1} \langle s(y) G(y)^{n_B-1} \rangle \sum_{j=2}^{n_B} \epsilon_j. \tag{S52}$$

Noticing that $\langle s(y) G(y)^{n_B-1} \rangle = \mathrm{Cov}\left(s(y), G(y)^{n_B-1}\right)$ since $\langle s(y) \rangle = 0$ and applying the covariance inequality

$$\left[\mathrm{Cov}(s(y), G(y)^{n_B-1})\right]^2 \leq \mathrm{Var}(s(y)) \times \mathrm{Var}(G(y)^{n_B-1}), \tag{S53}$$

we get

$$\langle s(y) G(y)^{n_B-1} \rangle^2 \leq \mathcal{I}_Y \left(\frac{1}{2n_B - 1} - \frac{1}{n_B^2} \right).$$

Note that $\mathrm{Var}(s(y)) = \mathcal{I}_y$ and that $\mathrm{Var}(G(y)^{n_B-1}) = \langle G(y)^{2n_B-2} \rangle - \langle G(y)^{n_B-1} \rangle^2$.

Therefore, selection fidelity is bounded from above as follows

$$\xi_Y \leq \frac{1}{n_B} + \frac{1}{n_B \sqrt{2n_B - 1}} \sqrt{\mathcal{I}_Y(E_{b1})} \sum_{i=2}^{n_B} (E_{b1} - E_{bi}). \tag{S54}$$

- When a readout distribution is inert to changes in affinity, $\mathcal{I}_Y = 0$, then there is at most a probability of $1/n_B$ to rank the cell with the highest affinity at the top.
- Setting $n_B = 2$, we obtain the fidelity bound for distinguishing two cells (main Eq. 4):

$$\xi_Y \leq \mu_{\rm cdf} + \sigma_{\rm cdf}\sqrt{\mathcal{I}_Y}\,\epsilon + \mathcal{O}\big(\epsilon^2\big). \tag{S55}$$

  Here $\mu_{\rm cdf} = \langle G(y)\rangle$ and $\sigma_{\rm cdf} = \sqrt{{\rm Var}(G(y))}$. This upper bound applies to continuous and discrete distributions.

B. Examples: validity of the upper bound

To demonstrate the validity of the upper bound (Eq.S55), we calculate the selection fidelity for a few typical readout distributions using $\xi_Y = {\rm Prob}(y_1 > y_2) = \int_{-\infty}^{\infty} dy_1 P(y_1) \int_{-\infty}^{y_1} dy_2 P(y_2)$.

a. Binary distribution If the readout follows a binary distribution $P(y; E_b) = \delta(y)(1-\eta(E_b)) + \delta(1-y)\eta(E_b)$, where y is an indicator of success ($y = 1$) or failure ($y = 0$), we find

$$\xi_Y = \eta(1-\eta) + \eta\frac{\partial \eta}{\partial E_b}\epsilon + \mathcal{O}\big(\epsilon^2\big). \tag{S56}$$

This is exactly the fidelity bound for pairwise discrimination:

$$\xi_Y = \mu_{\rm cdf} + \sigma_{\rm cdf}\sqrt{\mathcal{I}_Y}\,\epsilon + \mathcal{O}\big(\epsilon^2\big). \tag{S57}$$

Here we have used the following results

$$\mathcal{I}_Y = \frac{1}{(1-\eta)\eta}\left(\frac{\partial \eta}{\partial E_b}\right)^2, \quad \mu_{\rm cdf} = \eta(1-\eta), \quad \sigma_{\rm cdf} = \eta\sqrt{\eta(1-\eta)}.$$

b. Exponential distribution When the readout follows an exponential distribution $P(y; E_b) = \frac{1}{\mu(E_b)} e^{-y/\mu(E_b)}\Theta(y)$ where $\Theta(\cdot)$ denotes the step function, the selection fidelity reads

$$\xi_Y = \frac{1}{2} + \frac{1}{4}\sqrt{\mathcal{I}_Y}\,\epsilon + \mathcal{O}\big(\epsilon^2\big). \tag{S58}$$

Specifically, we find that

$$\xi_Y = \frac{\mu(E_{b1})}{\mu(E_{b1}) + \mu(E_{b2})} = \frac{1}{2} + \frac{1}{4\mu(E_{b1})}\frac{{\rm d}\mu}{{\rm d}E_b}\bigg|_{E_{b1}}\epsilon + \mathcal{O}\big(\epsilon^2\big)$$

and that

$$\mathcal{I}_Y = \frac{1}{\mu^2}\left(\frac{{\rm d}\mu}{{\rm d}E_b}\right)^2\bigg|_{E_{b1}}.$$

c. Gaussian distribution Consider that the readout distribution is Gaussian with a fixed variance $y \sim \mathcal{N}(\mu(E_b), \sigma)$. Direct calculation yields

$$\xi_Y = \frac{1}{2} + \frac{1}{2\sqrt{\pi}}\sqrt{\mathcal{I}_Y}\,\epsilon + \mathcal{O}\big(\epsilon^2\big). \tag{S59}$$

Hence, the selection fidelity of these readout distributions is indeed bounded from above by $\frac{1}{2} + \frac{1}{2\sqrt{3}}\sqrt{\mathcal{I}_Y}\,\epsilon + \mathcal{O}\big(\epsilon^2\big)$ that follows from Eq. S54.

C. Generalization to top-K ranking fidelity

One can generalize selection fidelity to the probability of ranking the top-K high affinity cells above the rest. Assuming that $E_{b1} > E_{b2} > \cdots > E_{bn_B}$ and denoting the readout of B cell i with y_i, we have

$$\begin{aligned}\xi_Y^{(K)} &\equiv {\rm Prob}(y_1, y_2, \cdots, y_K > y_{K+1}, \cdots, y_{n_B}) \\ &= \prod_{i=1}^{K} {\rm Prob}(y_i > y_{K+1}, \cdots, y_{n_B})\end{aligned} \tag{S60}$$

As long as antigen extraction by individual cells is independent, each term in the product is what we calculated above for the top-1 ranking fidelity (Eq. S54). That is,

$$\xi_{Y,i}^{(1)} \leq \frac{1}{n_B - K + 1} + \frac{1}{(n_B - K + 1)\sqrt{2(n_B - K + 1) - 1}} \sqrt{\mathcal{I}_Y} \sum_j \epsilon_{ij} + \sum_j \mathcal{O}(\epsilon_{ij}^2)$$

where $\epsilon_{ij} = E_{bi} - E_{bj}$ with j running from $K + 1$ to n_B.

Thus, to the leading order in ϵ, we obtain an upper bound for the top-K selection fidelity

$$\xi_Y^{(K)} \leq \left(\frac{1}{n_B - K + 1}\right)^K + \left(\frac{1}{n_B - K + 1}\right)^{K-1} \frac{1}{(n_B - K + 1)\sqrt{2(n_B - K + 1) - 1}} \sqrt{\mathcal{I}_Y} \sum_i \sum_j \epsilon_{ij} + \sum_i \sum_j \mathcal{O}(\epsilon_{ij}^2). \tag{S61}$$

Therefore, despite different prefactors of $\sqrt{\mathcal{I}_Y}$ due to differences in the readout distribution and the number of top-ranked cells, the square-root scaling of the fidelity bound with the Fisher information remains unchanged.

V. OPTIMAL CLUSTER SIZE

To determine the contact pattern that maximizes the total information an n-discriminator can acquire, we consider a total of N_0 complexes divided into clusters of equal size N, each subject to a pulling force of magnitude F. Assuming that rebinding is negligible, each cluster dissociates through a succession of rupture events. In this mean-field description with homogeneous cluster size N and force magnitude F, the lower bound of the total information is given by

$$\tilde{\mathcal{I}}_{n,\text{tot}}(N, F) = \frac{N_0}{N} \tilde{\mathcal{I}}_n(N, F) = \beta^2 \frac{N_0}{N} \sum_{i=1}^{N} \frac{e^{\beta(\Delta E_0 - F\Delta x/i)}}{\left[1 + e^{\beta(\Delta E_0 - F\Delta x/i)}\right]^2}. \tag{S62}$$

The optimal cluster size N^* maximizes the total information extracted under a given force per cluster, that is

$$N^* \equiv \arg\max_N \tilde{\mathcal{I}}_{n,\text{tot}}(N, F). \tag{S63}$$

For moderately large clusters, we can approximate the sum over rupture events with an integral, which gives

$$\tilde{\mathcal{I}}_{n,\text{tot}} \approx \beta^2 N_0 \int_0^1 \frac{e^{\beta(\Delta E_0 - F\Delta x/Nu)}}{\left[1 + e^{\beta(\Delta E_0 - F\Delta x/Nu)}\right]^2} du, \tag{S64}$$

where the continuous variable $u = i/N$ denotes the fraction of persisting 3-body complexes. Note that for a given affinity gap $\Delta E_0 = E_b - E_a$ and a bond length difference $\Delta x = x_b - x_a$, $\tilde{\mathcal{I}}_{n,\text{tot}}$ is a function of $F\Delta x/N\Delta E_0$ only. Since the largest contribution to the integral comes from the range of u that gives a small affinity gap such that $\beta(\Delta E_0 - F\Delta x/Nu) \simeq 0$, we Taylor expand the integrand and limit the integration range to obtain

$$\tilde{\mathcal{I}}_{n,\text{tot}} \approx \beta^2 N_0 \int_{u_{\min}}^{u_{\max}} \left[\frac{1}{4} - \frac{1}{16}(\beta\Delta E_0)^2 \left(1 - \frac{F\Delta x}{N\Delta E_0 u}\right)^2\right] du. \tag{S65}$$

The choice of $u_{\min}$ and $u_{\max}$ ensures a non-negative integrand (see Fig. S7A):

$$u_{\min} = \frac{\beta\Delta E_0}{\beta\Delta E_0 + 2} \frac{F\Delta x}{N\Delta E_0} < 1, \qquad u_{\max} = \begin{cases} \min\left(1, \frac{\beta\Delta E_0}{\beta\Delta E_0 - 2} \frac{F\Delta x}{N\Delta E_0}\right), & \text{if } \beta\Delta E_0 > 2 \\ 1, \quad \text{otherwise.} \end{cases} \tag{S66}$$

Denoting $c = F\Delta x/(N\Delta E_0)$, the optimal cluster size can be found from $\frac{\mathrm{d}\tilde{\mathcal{I}}_{n,\text{tot}}}{\mathrm{d}c} = 0$ to be given by

$$N^* \approx \frac{F\Delta x}{c^*(\Delta E_0)\Delta E_0} \tag{S67}$$

where $c^*(\Delta E_0)$ is the smaller solution to the following equation

$$ce^{-c} = \left(1 + \frac{2}{\beta\Delta E_0}\right) e^{-(1+2/\beta\Delta E_0)}. \tag{S68}$$

Fig. S7B plots the total information as a function of c, comparing the approximation to the exact value, which makes clear that the smaller root should be chosen. Fig. S7C shows that the analytical solution of c^* agrees well with the numerical value that maximizes the total information. As ΔE_0 increases, $c^*(\Delta E_0)$ rises from zero and approaches 1 asymptotically.

VI. INFLUENCE OF REBINDING ON INFORMATION EXTRACTION

We now examine the effect of rebinding on information acquisition and show that frequent rebinding greatly enhances the performance of a τ-discriminator at the expense of extraction speed, but has little influence on an n-discriminator.

A deterministic description of the extraction dynamics provides intuition in cases where rebinding can stabilize finite clusters at steady state. The cluster size m evolves over time according to the kinetics of binding and (force-modulated) unbinding

$$\frac{\mathrm{d}m}{\mathrm{d}t} = -mk_0\left[e^{-\beta(E_a - \frac{F}{m}x_a)} + e^{-\beta(E_b - \frac{F}{m}x_b)}\right] + (N-m)k_{\text{on}}. \tag{S69}$$

At steady state, a bifurcation point marks the minimum rebinding rate that allows finite clusters to persist for a given affinity gap (Fig. S6A, symbol). Above the bifurcation point, cluster lifetime steeply rises with the on rate (Fig. S6B) and the FI is strongly enhanced due to repeated unbinding and rebinding of individual complexes (Fig. S6C, red region). This behavior instantiates the idea that nonequilibrium sensing lowers the estimation error by increasing the number of measurements per receptor, while compromising the decision speed.

In the limit of frequent rebinding ($k_{\text{on}} \gg k_{a,i} + k_{b,i}$ for all i), the stochastic system stays near the deterministic steady state for long, until a rare, large fluctuation drives the cluster toward complete dissociation. In this regime, cluster lifetime assumes an exponential distribution (Fig. S2B) and the FI becomes independent of k_{on}:

$$\tilde{\mathcal{I}}_\tau \approx \beta^2 \left(\sum_{i=1}^{N} \frac{1}{1+e^{\beta\Delta E_i}}\right)^2, \tag{S70}$$

where $\Delta E_i = E_b - E_a - F(x_b - x_a)/i$. Thus, under weak force or large affinity gap and frequent rebinding, $\mathcal{I}_\tau \sim N^2$, that is, information exhibits a stronger scaling with system size compared to $\mathcal{I}_\tau \sim (\ln N)^2$ under weak rebinding (Fig. S6B). However, rebinding does not alter the affinity dependence at high affinities; we still get $\mathcal{I}_\tau \propto e^{-2\beta E_b}$ when $\beta\Delta E_i \gg 1$.

In contrast, rebinding has a very limited impact on an n-discriminator. This is because the primary route by which rebinding can alter the extraction level is through closing the bonds that break before the system reaches the equilibrium cluster size; these bonds later reopen under a different load after the equilibrium state is reached. This effect diminishes at higher on rates, because fewer bonds break before the cluster reaches the equilibrium size. This is why the moderate effect of rebinding on $\mathcal{I}_n$ is only detectable at intermediate k_{on} near the bifurcation where sensitivity to perturbations is most pronounced (Fig. S6D). Note that, both the scaling with N and the dependence on affinity remain unchanged despite frequent rebinding. Therefore, physical extraction robustly outperforms lifetime measurement, especially at high affinities.

VII. BELL'S PHENOMENOLOGICAL MODEL VS LANDSCAPE MODEL OF BOND RUPTURE

Depending on the problem to solve and the available observations, one can make different choices of the model off-rates. To capture known effects of force on bond lifetime while lessening the computational load of solving the master equation for the full readout distributions at relevant cluster sizes (up to $N \sim 1000$), we choose the simplest form — Bell's phenomenological model of bond rupture[2]. In Bell's model, force per bond f is linearly coupled to the bond extension at rupture, which enters the off-rates only through the Arrhenius factor, lowering the free energy barrier to bond dissociation (Eq. 2 in the main text). Kramers theory based on a free energy landscape, on the other hand, provides a more accurate relationship between bond lifetime and applied force, as it accounts for force-induced deformation of the free energy surface, which not only lowers the activation barrier but also displaces the bound state and the saddles, quantitatively modifying the off-rates and the extraction probability[1]. Below we demonstrate that using a landscape model for bond rupture does not alter in any qualitative way our central results — the affinity dependent information content of two complementary recognition modes/discriminators.

For simplicity, we consider the scenario of independent complexes in which force per bond remains constant. Following the same procedure as described in the SI section IIIA, we can calculate Fisher information contained in both affinity readouts. For the distribution of cluster lifetime, $P_\tau(t)$, the curvature of the log-likelihood is given by

$$\frac{\partial^2 \ln P_\tau(t)}{\partial E_b^2} = -\Big(\frac{\partial\lambda}{\partial E_b}\Big)^2\Big[\frac{1}{\lambda^2} + (N-1)\frac{t^2e^{-\lambda t}}{(1-e^{-\lambda t})^2}\Big] + \frac{\partial^2\lambda}{\partial E_b^2}\Big[\frac{1}{\lambda} - t + (N-1)\frac{te^{-\lambda t}}{1-e^{-\lambda t}}\Big],$$

where $\lambda = k_a + k_b$ represents the 3-body rupture rate. Compared to the expression due to Bell's model (Eq. S31), the only differences are that $(\partial\lambda/\partial E_b)^2$ replaces $(\beta k_b)^2$ and that $\partial^2\lambda/\partial E_b^2$ replaces $\beta^2 k_b$. It follows that, for large N,

Fisher information contained in τ reads

$$\mathcal{I}_\tau \approx \frac{1}{\lambda^2}\left(\frac{\partial\lambda}{\partial E_b}\right)^2(\ln N)^2. \tag{S71}$$

Note that the scaling in N remains unchanged. Since the distribution of extraction level retains the same form

$$P(n) = \binom{N}{n}\frac{k_a^n k_b^{N-n}}{(k_a+k_b)^N}, \tag{S72}$$

so does the associated Fisher information

$$\mathcal{I}_n = -\sum_{n=0}^{N} P(n)\frac{\partial^2 \ln P(n)}{\partial E_b^2}. \tag{S73}$$

Nonetheless, the specific dependence of the off-rates (k_a, k_b) on BCR affinity E_b depends on the choice of landscape models. In general, the off-rate of the tugging interaction may depend on the intrinsic properties of the tethering interaction and vice versa. That is, one can express the off-rates in landscape models as $k_a(E_a, E_b, x_a, x_b, f)$ and $k_b(E_a, E_b, x_a, x_b, f)$, as opposed to those in Bell's model as $k_a(E_a, x_a, f)$ and $k_b(E_b, x_b, f)$. This is because the antigen molecule, subject to frictional force, couples the bond extension on either side.

To obtain concrete results of Fisher information from Eqs.S71 and S73, we consider two forms of the free energy surface — cusp-harmonic and linear-cubic — which are typical and suitable choices for interpreting single-molecule pulling experiments. Specifically, the off-rates due to a cusp-harmonic potential are given by[1]

$$\begin{aligned} k_a &= 2\sqrt{\beta E_a}E_a\left(1-\frac{f x_a}{2E_a}\right)\frac{1}{\gamma_a\sqrt{\pi}x_a^2}e^{-\beta E_a\left(1-\frac{f x_a}{2E_a}\right)^2} \\ k_b &= 2\sqrt{\beta E_b}E_b\left(1-\frac{f x_b}{2E_b}\right)\frac{\gamma_a+\gamma_b}{\gamma_a\gamma_b\sqrt{\pi}x_b^2}e^{-\beta E_b\left(1-\frac{f x_b}{2E_b}\right)^2} \end{aligned} \tag{S74}$$

Here γ_a and γ_b are frictional coefficients (of dissipative forces acting on the antigen and BCR molecules, respectively) that set the relaxation timescale. On the other hand, a linear-cubic potential yields off-rates of a more complex form[1]

$$\begin{aligned} k_a &= \frac{\kappa_a}{\pi\gamma_a}\left\{1+\frac{\gamma_b}{\gamma_a}\left(1-\frac{\kappa_a}{\kappa_b}\right)+\sqrt{\left[1+\frac{\gamma_b}{\gamma_a}\left(1-\frac{\kappa_a}{\kappa_b}\right)\right]^2+4\frac{\gamma_b\kappa_a}{\gamma_a\kappa_b}}\right\}^{-1} e^{-\beta E_a(1-f/f_a)^{3/2}} \\ k_b &= \frac{\kappa_b}{\pi\gamma_b}\left\{1-\frac{\kappa_b}{\kappa_a}\left(1+\frac{\gamma_b}{\gamma_a}\right)+\sqrt{\left[1-\frac{\kappa_b}{\kappa_a}\left(1+\frac{\gamma_a}{\gamma_b}\right)\right]^2+4\frac{\kappa_b\gamma_a}{\kappa_a\gamma_b}}\right\}^{-1} e^{-\beta E_b(1-f/f_b)^{3/2}} \end{aligned} \tag{S75}$$

Here $f_a = 3E_a/2x_a$, $f_b = 3E_b/2x_b$, $\kappa_a = \frac{4f_a}{x_a}\sqrt{1-f/f_a}$ and $\kappa_b = \frac{4f_b}{x_b}\sqrt{1-f/f_b}$.

In Fig. S8, we plot the total Fisher information as a function of effective BCR affinity, like in Fig. 2B in the main text, for both readouts (columns) under different choices of the potential (rows). Clearly, using landscape models yields the same trend of affinity dependence as for the Bell's model over a physiological force range; modest deviations reflect the effect of affinity and force dependent entropic factors absent from the Bell's model. Moreover, the specific form of the potential function makes little difference. Therefore, the distinctive behaviors of the two discriminators depend not much on the particular choice of the bond-rupture model, but on the distinct nature of the readouts.

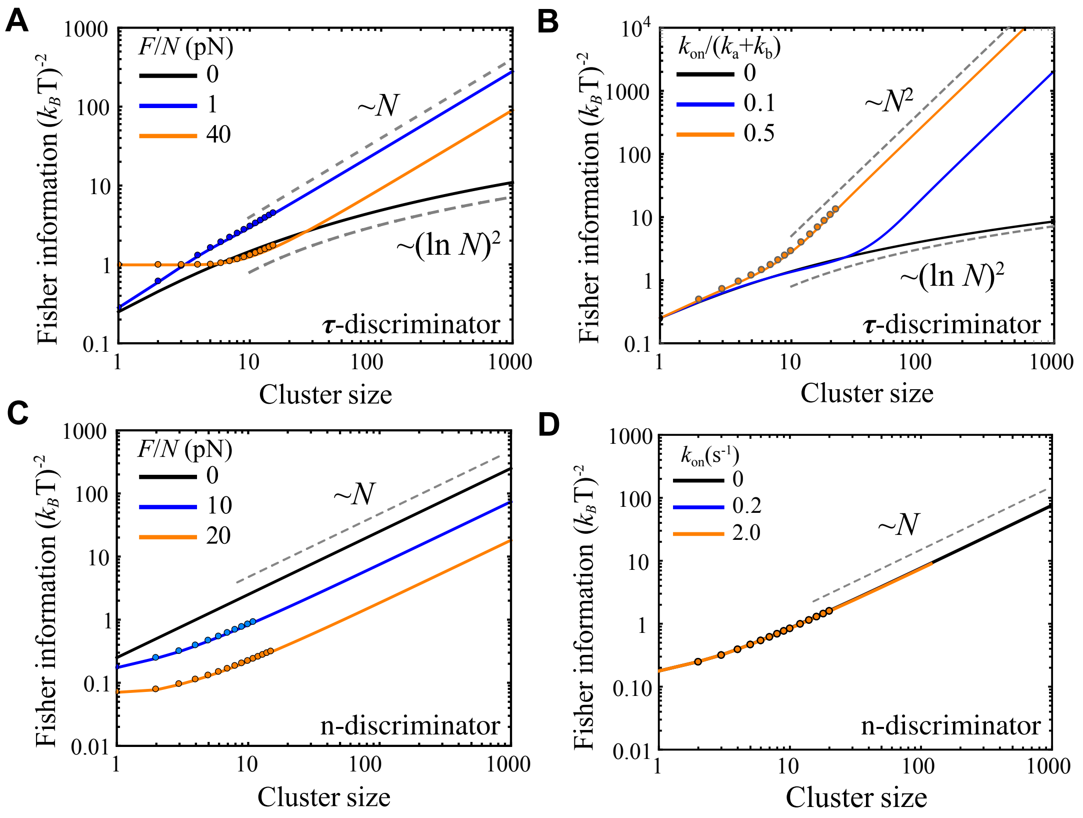

FIG. S1. **Load sharing and rebinding alter the scaling of Fisher information with cluster size in a readout-dependent manner.** (A, B) $\tilde{\mathcal{I}}_\tau$ as a function of cluster size N under a shared load (A) or with finite rebinding (B). (C, D) $\tilde{\mathcal{I}}_n$ as a function of cluster size N under a shared load (C) or with finite rebinding (D). While load sharing and rebinding alter the scaling relationship of a τ-discriminator, an n-discriminator retains a linear scaling with N. In all panels, we fix force per bond F/N when varying the cluster size N. Black curves represent the reference case without load sharing or rebinding. Dashed lines indicate the scaling relationship at large N. Curves are obtained from calculations in Section II. Symbols result from numerically solving Eq. S3 (τ-discriminator) or Eq. S16 (n-discriminator). We set $k_{\rm on} = 0$ in panels A and C, $F = 0$ in panel B and $F/N = 10$pN in panel D. $k_{a0} = k_{b0} = 1s^{-1}, x_a = 1.5nm, x_b = 2$nm.

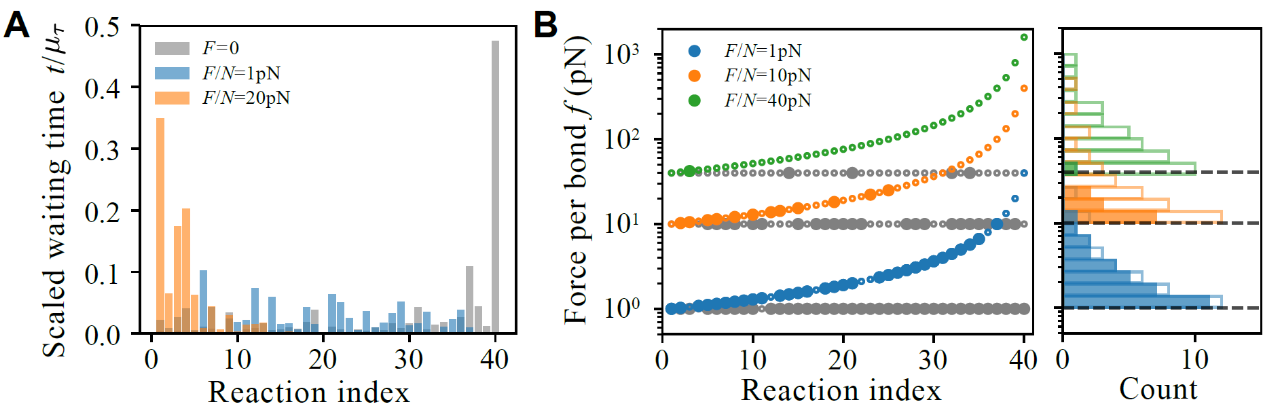

FIG. S2. **Load sharing strongly influences the distribution of waiting time and the success rate of extraction.** Each set of data comes from an independent simulation; we plot the quantities against the reaction index, which labels the rupture events in the order of occurrence. (A) Histogram of the waiting time (scaled by the mean cluster lifetime) between consecutive rupture events. When force is very weak (grey) or very strong (orange), the last or first few events in large part account for the cluster lifetime. Under a modest load (blue), waiting times are similar and many events contribute to cluster lifetime. (B) Trajectory of force per bond. As the reactions proceed, bonds break successively, and the force acting on each remaining bond increases (colored symbols). Filled circles indicate successful antigen extraction. Grey symbols show, for comparison, a typical sequence of rupture events under independent force (i.e. force per bond remains constant). Filled circles again mark success of extraction. The histograms on the right show the corresponding distributions of force per bond due to each sequence of rupture events. The filled portion indicates the fraction of events leading to antigen extraction. As the initial force per bond F/N increases, the chance of extraction falls. Parameters: $N = 40, k_{a0} = 1s^{-1}, k_{b0} = 0.13s^{-1}$.

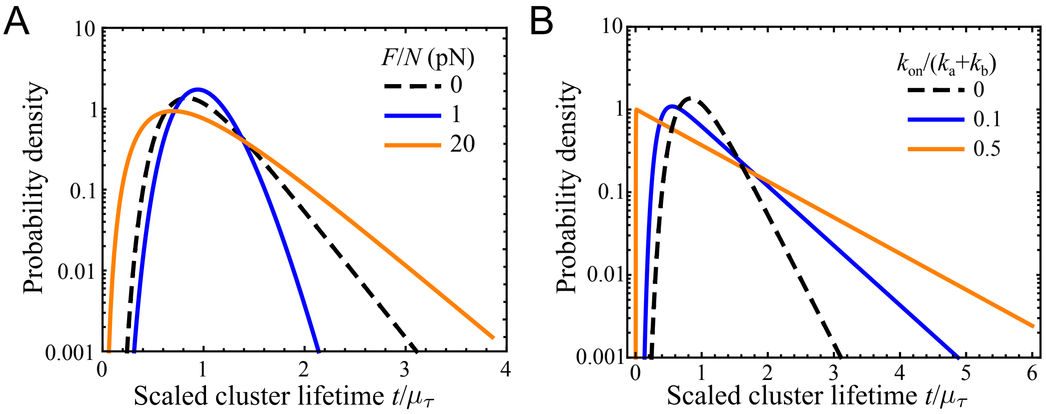

FIG. S3. **Load sharing and rebinding alter the nature and statistics of the cluster lifetime distribution.** In both panels, the dashed line represents the reference distribution in the absence of force and without rebinding. It follows an extreme value distribution, since the most long-lived bond determines the cluster lifetime. Curves are obtained by numerically solving the forward master equation Eq. S3. The cluster lifetime (x-axis) is scaled by its mean value μ_τ. (A) Strong pulling yields a near-exponential distribution (orange) since breaking of the first few bonds follows a Poisson process. In contrast, a modest shared load produces a Gaussian-like distribution (blue), because many contributing events are spaced by similar waiting times. (B) As rebinding becomes more frequent, cluster lifetime approaches an exponential distribution, because frequent rebinding generates an effective barrier against cluster dissociation. Only occasionally, noise drives the system over the barrier. That is, cluster dissociation becomes a noise-driven rare event. Parameters: $N = 20, k_{a0} = k_{b0} = 1\text{s}^{-1}$.

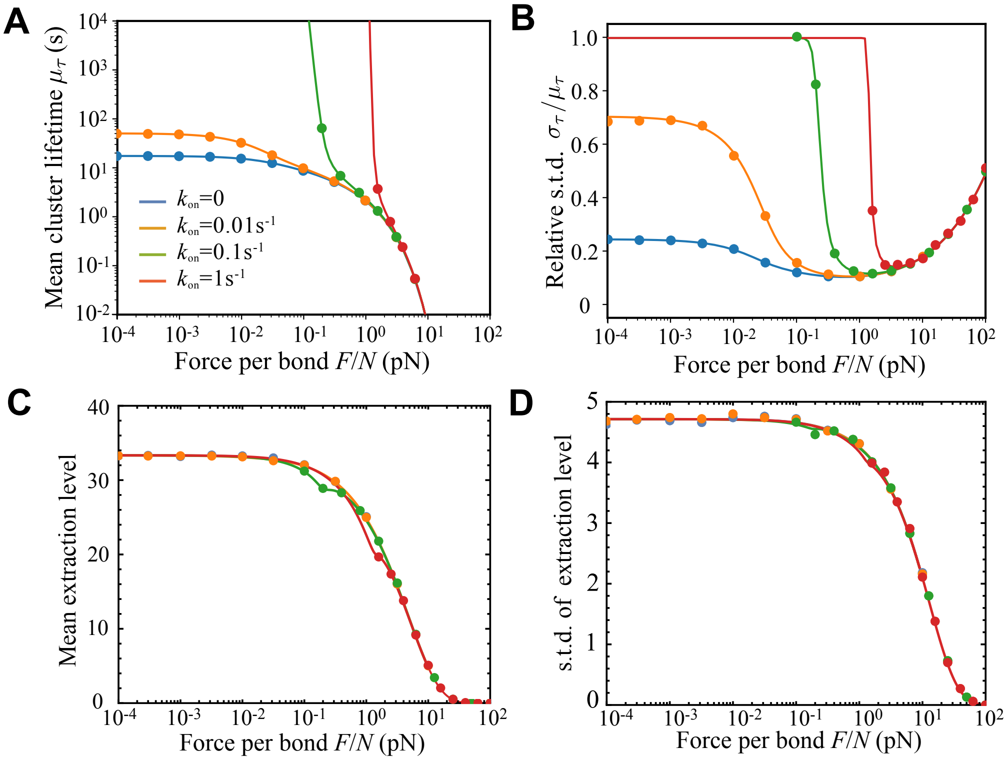

FIG. S4. **Statistics of affinity readouts resulting from antigen extraction under a shared load with finite rebinding.** (A, B) The mean μ_τ and relative standard deviation σ_τ/μ_τ of the cluster lifetime are plotted as a function of the magnitude of force per bond F/N. (A) Modest rebinding strongly enhances cluster lifetime when force is weak. Moderately strong force can overcome rebinding; typically, a cluster dissociates within a second when the force per bond is only a few pN. The relative variation of cluster lifetime is most effectively suppressed by a modest force per bond of roughly 1pN. Curves are calculated from Eq. S9 and Eq. S11; symbols are results of stochastic simulations of Eq. S1. (C, D) The mean and standard deviation of extraction level n_{Ag} show similar dependence on pulling strength, both starting to decline as the force per bond approaches 1pN. Rebinding has little influence on extraction level; only at intermediate F/N near the bifurcation point is a small dip visible. Curves are obtained from numerically solving Eqs. S21-S23. Symbols are results of stochastic simulations of Eq. S1, averaged over 5000 runs each. For both readouts, the agreement between theory and simulation is excellent. Parameters: $N = 100, k_{a0} = 0.1\mathrm{s}^{-1}, k_{b0} = 0.2\mathrm{s}^{-1}$.

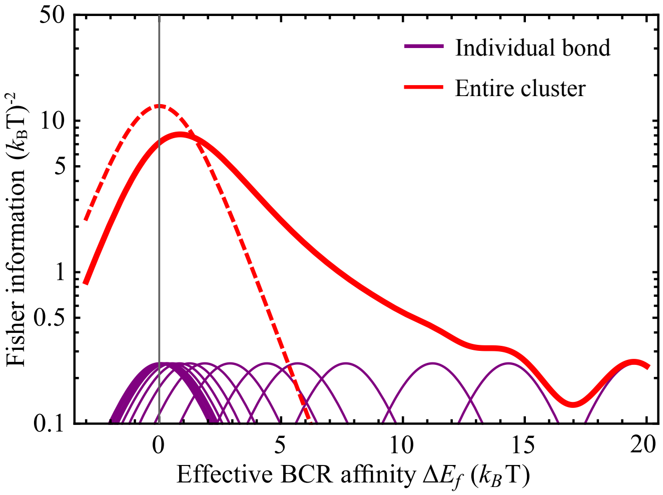

FIG. S5. **Expansion of discrimination range under moderate pulling due to partially overlapping sensitive windows.** Red dashed line: Under independent extraction events, Fisher information $\tilde{\mathcal{I}}_n$ peaks at zero effective BCR affinity, i.e. when the affinity gap between BCR and antigen tether is closed for every bond in the cluster. Red solid line: Under a shared load, a cell can acquire information about BCR affinity over a wider range, because sensitive windows of consecutive bonds (purple humps) no longer coincide; the sensitive window for a bond that breaks when a total of i bonds remain closed is centered at $E_b = E_a + F(x_b - x_a)/i$. Hence, as more bonds break, the spacing between adjacent windows increases. $N = 50, k_{a0} = 1\text{s}^{-1}, k_{\text{on}} = 0, x_a = 1.5\text{nm}, x_b = 2.0\text{nm}, F = 500\text{pN}$.

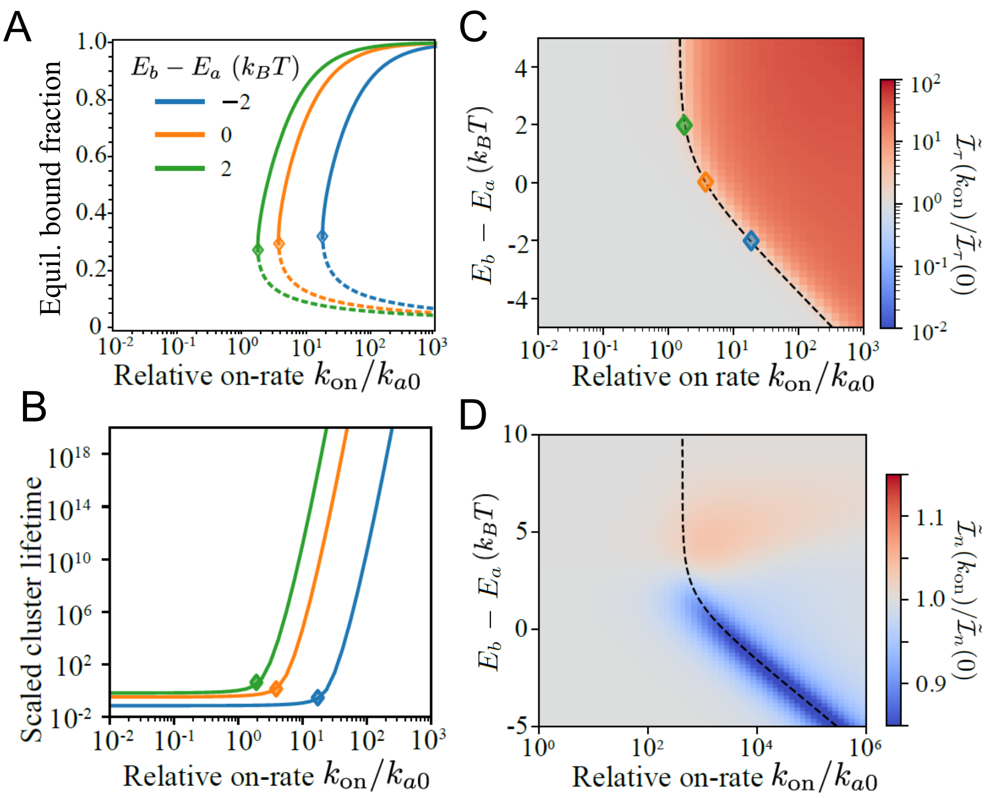

FIG. S6. **Rebinding increases Fisher information in cluster lifetime at the expense of extraction speed but has little influence on extraction level.** (A) Equilibrium cluster sizes in the deterministic picture. Curves are steady-state solutions to Eq. S69, showing the stable (solid) and unstable (dashed) branches. Symbols mark the bifurcation point – the rebinding rate at which an equilibrium state first appears. (B) Mean cluster lifetime $\bar{\tau}$ (Eq. S9) scaled by $1/k_{a0}$ as a function of the relative rebinding rate, with symbols indicating the bifurcation points. (C) Scaled Fisher information in cluster lifetime, $\tilde{\mathcal{I}}_\tau(k_{\rm on})/\tilde{\mathcal{I}}_\tau(0)$, as a function of the relative on rate, $k_{\rm on}/k_{\rm a0}$, and the affinity gap, $E_b - E_a$. The dashed line indicates the onset of bifurcation. $F/N = 1$pN. Above bifurcation, cluster lifetime steeply increases (panel B); the resulting numerous unbinding and rebinding events strongly enhance the information content of cluster lifetime (panel C, red region). (D) Scaled Fisher information in extraction level, $\tilde{\mathcal{I}}_n(k_{\rm on})/\tilde{\mathcal{I}}_n(0)$. $F/N = 10$pN. Other parameters: $x_a = 1.5$nm, $x_b = 2$nm, $N = 30$.

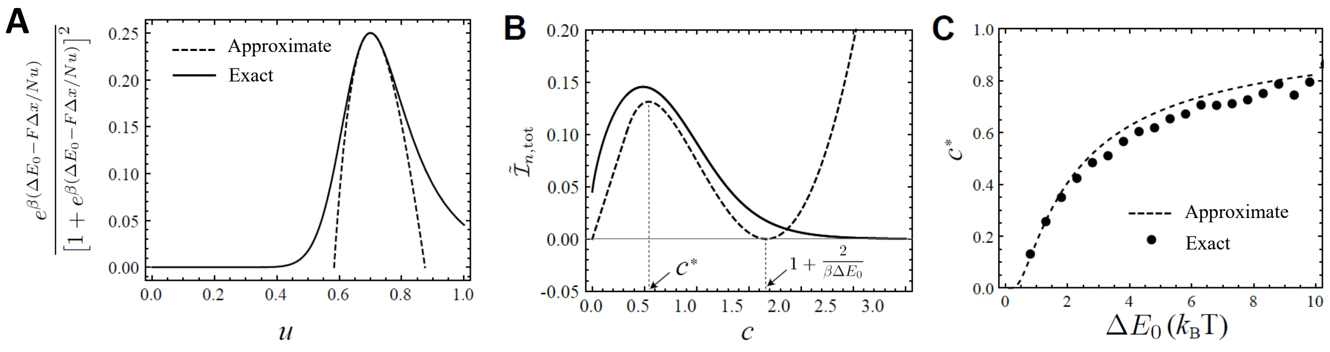

FIG. S7. **Affinity dependence of the optimal cluster size.** (A) Exact (solid line) and approximate (dashed line) forms of the integrand in Eq. S64 for estimating the total Fisher information $\tilde{\mathcal{I}}_{n,\rm tot}$ contained in the extraction level. Curves are generated with $\Delta E_0 = 3k_{\rm B}T$ and $F\Delta x/N\Delta E_0 = 0.3$. (B) $\tilde{\mathcal{I}}_{n,\rm tot}$ as a function of the dimensionless variable $c = F\Delta x/N\Delta E_0$. The smaller ($c^*$) of the two possible solutions (arrows) should be chosen, as it maximizes total information. $\Delta E_0 = 3k_{\rm B}T$ is used. (C) c^* has a sublinear dependence on affinity gap $\Delta E_0 = E_b - E_a$, rising from zero and approaching one as ΔE_0 increases.

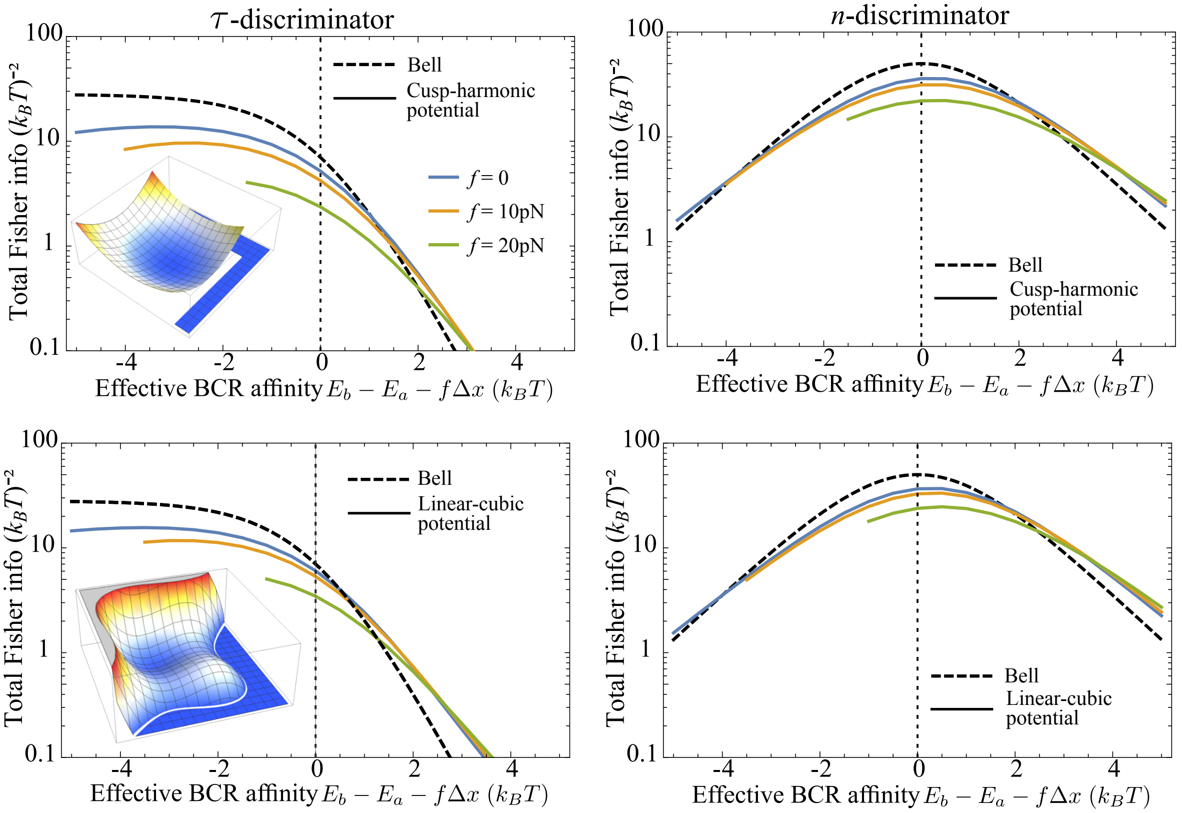

FIG. S8. **Fisher information under landscape models.** Total Fisher information encoded in cluster lifetime (left column) and antigen extraction level (right column) is shown as a function of the effective BCR affinity $E_b - E_a - f(x_b - x_a)$. We compare the results between Bell's model (dashed line) and landscape model at different magnitudes of force per complex (colored solid lines). We use the cusp-harmonic potential (upper row) and the linear-cubic potential (lower row), illustrated in the insets over the plane of bond extensions of the BCR-Ag and Ag-tether interactions. Since the expressions of the off-rates (Eqs. S74 and S75) are only valid for high activation barriers, we set a cutoff barrier height at $3k_{\mathrm{B}}T$. The qualitative behavior remains the same as in Fig. 2 of the main text. No rebinding. Independent complexes, each subject to a constant force of magnitude f. $N = 100, x_a = 1.5\mathrm{nm}, x_b = 2\mathrm{nm}, E_a = 10k_{\mathrm{B}}T$, $\gamma_a = \gamma_b = 10^{-5}\mathrm{Ns/m}$.

---

[1] Hongda Jiang and Shenshen Wang. Immune cells use active tugging forces to distinguish affinity and accelerate evolution. *Proceedings of the National Academy of Sciences*, 120(11):e2213067120, 2023.

[2] George I Bell. Models for the specific adhesion of cells to cells: a theoretical framework for adhesion mediated by reversible bonds between cell surface molecules. *Science*, 200(4342):618–627, 1978.
